# CRISPRi enables isoform-specific loss-of-function screens and identification of gastric cancer-specific isoform dependencies

**DOI:** 10.1186/s13059-021-02266-6

**Published:** 2021-01-26

**Authors:** Rebecca Davies, Ling Liu, Sheng Taotao, Natasha Tuano, Richa Chaturvedi, Kie Kyon Huang, Catherine Itman, Amit Mandoli, Aditi Qamra, Changyuan Hu, David Powell, Roger J. Daly, Patrick Tan, Joseph Rosenbluh

**Affiliations:** 1 Cancer Research Program and Department of Biochemistry and Molecular Biology, Biomedicine Discovery Institute, Monash University, Clayton, VIC 3800 Australia; 2 Program in Cancer and Stem Cell Biology, Duke-NUS Medical School, Singapore, 169857 Singapore; 3 Cancer Science Institute of Singapore, National University of Singapore, Singapore, 117599 Singapore; 4 Cancer Therapeutics and Stratified Oncology, Genome Institute of Singapore, Singapore, 138672 Singapore; 5 SingHealth/Duke-NUS Institute of Precision Medicine, National Heart Centre Singapore, Singapore, 169856 Singapore; 6 Cellular and Molecular Research, National Cancer Centre, Singapore, 169610 Singapore; 7 Singapore Gastric Cancer Consortium, Singapore, 119074 Singapore; 8 Functional Genomics Platform, Monash University, Clayton, VIC 3800 Australia; 9 Monash Bioinformatics Platform, Monash University, Clayton, VIC 3800 Australia

## Abstract

**Introduction:**

Genes contain multiple promoters that can drive the expression of various transcript isoforms. Although transcript isoforms from the same gene could have diverse and non-overlapping functions, current loss-of-function methodologies are not able to differentiate between isoform-specific phenotypes.

**Results:**

Here, we show that CRISPR interference (CRISPRi) can be adopted for targeting specific promoters within a gene, enabling isoform-specific loss-of-function genetic screens. We use this strategy to test functional dependencies of 820 transcript isoforms that are gained in gastric cancer (GC). We identify a subset of GC-gained transcript isoform dependencies, and of these, we validate CIT kinase as a novel GC dependency. We further show that some genes express isoforms with opposite functions. Specifically, we find that the tumour suppressor *ZFHX3* expresses an isoform that has a paradoxical oncogenic role that correlates with poor patient outcome.

**Conclusions:**

Our work finds isoform-specific phenotypes that would not be identified using current loss-of-function approaches that are not designed to target specific transcript isoforms.

**Supplementary Information:**

The online version contains supplementary material available at 10.1186/s13059-021-02266-6.

## Introduction

Transcript isoforms are a major source of bio-diversity and have a critical role in regulating biological responses. In mammalian cells, two main mechanisms drive the generation of transcript isoforms: alternative splicing and differential promoter usage. Alternative splicing is a post-transcriptional mechanism to generate transcript isoforms and uses the splicing machinery. Differential promoter usage is a mechanism by which alternative promoters within a gene drive the expression of different transcript isoforms [1]. Promoter usage is defined by measurable epigenetic promoter changes that include H3K4me3 and H3K27ac [2–4]. RNA-Seq in conjugation with ChIP-Seq profiling in human cells has shown that the majority of transcript isoforms are produced by differential promoter usage rather than alternative splicing [5]. Consistent with these observations, most genes in the human genome contain multiple promoters that have the ability to transcribe various isoforms [6, 7]. Although we do not fully understand how cells determine which promoter to use, selection of a particular promoter will lead to the expression of a specific transcript isoform with a unique function. Therefore, in addition to regulating when a gene is transcribed, promoters also regulate which isoforms are expressed.

Large-scale programs such as the FANTOM network use CAGE-Seq to measure the abundances of transcript isoforms in various tissue organisms and disease conditions [8]. These studies found that transcript isoforms are tissue and cell type-specific, and different isoforms are expressed or suppressed in different disease states. In cancer, we and others found that gain and loss of promoters is pervasive, resulting in the expression of hundreds of cancer-specific transcript isoforms [9, 10]. Indeed, some cancer associate isoforms such as *ALK* [11] and *RASA3* [10] were shown to play a role in cancer progression while other cancer-specific isoforms such as *ERBB2* were found to have a better prognostic value then canonical *ERBB2* expression [9]. Furthermore, in some cases, isoforms from the same gene could have unique and sometimes opposite functions. For example, the anti-apoptotic *BCL2L1* gene expresses two isoforms that have different protein products: BCL-xL which inhibits apoptosis and BCL-xS which promotes apoptosis [12]. Taken together, all the emerging data demonstrate the importance of transcript isoforms in regulating biological processes. Recognising the importance of isoforms, over the past years, there have been a large number of experimental approaches that have been developed to measure isoform abundances [2, 8]. The next step is to define the function of these transcript isoforms using gain- and loss-of-function studies. However, despite the dramatic progress we have made in functional genomics, approaches for isoform-specific functional screens are still lacking.

CRISPR technology has been rapidly transforming our ability to understand gene function in normal or disease conditions [13]. CRISPR knockout (CRISPRko) targets WT Cas9 to gene exons generating indels and missense mutations that ultimately result in suppressing the expression of the target gene [14]. Since many transcript isoforms share a large portion of their exons, it is challenging to design isoform-specific CRISPRko sgRNAs. CRISPRi uses a catalytically inactive Cas9 (dCas9) fused to a transcription repressor (KRAB) that when targeted to a promoter inhibits gene expression [15]. A major limitation in the use of CRISPRi is the requirement to identify the exact location of the transcriptional start site (TSS). In fact, others [16] and us [17] have shown that CRISPRi works only within a limited window from the TSS. Although these properties are challenging in CRISPRi screens aimed at identifying phenotypes associated with genes, in this case, CRISPRi enables highly specific differential targeting of promoter-driven isoforms. Here, we exploited this feature of CRISPRi and developed a CRISPRi sgRNA library that targets promoters that are gained or lost in GC and drive the expression of GC-associated transcript isoforms.

## Results

### Targeting of dCas9-KRAB to promoters results in suppressing the expression of specific isoforms

**Fig. 1:**
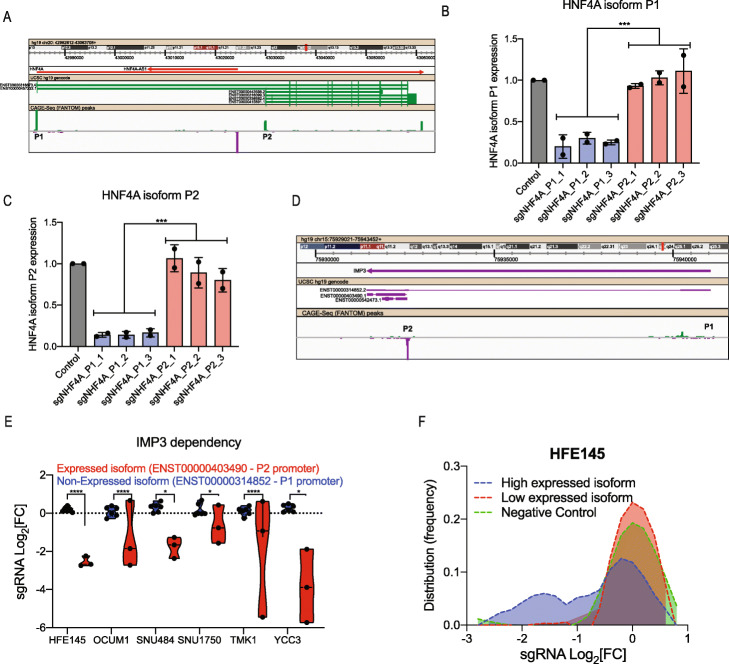
CRISPRi as a tool for inhibition of specific promoter-driven transcript isoforms. **a** Structure of the *HNF4A* gene. Isoforms P1 and P2 are marked. CAGE-Seq peaks from the FANTOM project [19] are shown in the bottom panel. **b** qRT-PCR quantification of *HNF4A* transcript P1 following CRISPRi-mediated suppression of transcript P1 or P2. Data is shown as mean ± SD, *n* = 2. pValue is calculated using two-tailed unpaired *t* test (***p ≤ 0.001). **c** qRT-PCR quantification of *HNF4A* transcript P2 following CRISPRi-mediated suppression of transcript P1 or P2. Data is shown as mean ± SD, *n* = 2. pValue is calculated using two-tailed unpaired *t* test (***p ≤ 0.001). **d** Structure of the *IMP3* gene. Isoforms P1 and P2 are marked. CAGE-Seq peaks from the FANTOM project are shown in the bottom panel. **e** Violin plot showing IMP3 dependency following CRISPRi-mediated suppression of different isoforms in GC cell lines. Dots represent individual sgRNAs targeting the indicated *IMP3* transcript isoform. pValue is calculated using two-tailed unpaired *t* test (****p ≤ 0.0001, *p ≤ 0.05). **f** Distribution of sgRNAs targeting different transcript isoforms of 55 pan cell-essential transcripts. Green, negative control sgRNAs. Purple, sgRNAs targeting the highest expressed (based on RNA-Seq) transcript isoform. Red, sgRNAs targeting the low expressed (based on RNA-Seq) transcript isoform

Our previous work using tiling sgRNA libraries found that a critical consideration in the design of effective CRISPRi sgRNAs is the distance from the TSS [17]. Others and us have shown that CRISPRi-induced phenotypes are only observed when dCas9-KRAB is targeted to a defined region that is located ± 150 bp from the TSS [16, 18]. Based on these observations, we hypothesised that targeting of dCas9-KRAB to a specific promoter within a gene will enable isoform-specific suppression. To test this hypothesis, we used the *HNF4A* gene which expresses two transcript isoforms (P1 and P2 in Fig. 1a). Both of these *HNF4A* isoforms show a CAGE-Seq peak (Fig. 1a, bottom panel) indicating that both of these transcripts are expressed. Following infection of YCC3 cells with sgRNAs targeting *HNF4A* promoter P1 or promoter P2, we measured the expression of these two *HNF4A* transcript isoforms using isoform-specific primers. We found that sgRNAs targeting promoter P1 had an effect only on *HNF4A* isoform 1 expression (Fig. 1b), and sgRNA targeting promoter P2 inhibited only *HNF4A* isoform 2 expression (Fig. 1c).

To assess the ability of CRISPRi to induce isoform-specific phenotypes, we developed a pooled sgRNA library targeting 55 pan cell-essential genes (defined as genes that had a lethal effect in more than 90% of cell lines in DepMap [20]). For each of these 55 genes, we used high-coverage RNA-Seq in 4 cell lines and the transcript-specific Salmon algorithm [21] (Additional file 2: Table S1) to identify the most highly expressed isoform as well as the second most abundant transcript. Based on these annotations, we designed a pooled sgRNA library (Additional file 2: Table S2) and used this library to transduce 5 GC cell lines and one normal immortalised gastric cell line (HFE145). Genomic DNA extracted 21 days post-infection was used for sequencing and quantification of sgRNA abundances. Using this dataset, we found that only sgRNAs targeting the most abundant transcript had the expected anti-proliferation effect. For example, *IMP3* is a pan-essential gene that contains two known promoters (P1 and P2 in Fig. 1d). CAGE-Seq analysis from the FANTOM5 consortium [8] shows that different from *HNF4A* (Fig. 1a), only the P2 promoter of *IMP3* is active while P1 has only residual transcriptional activity (Fig. 1d). Consistent with these measurements, we found that sgRNAs targeting dCas9-KRAB to the P2 promoter and not the P1 promoter had the expected lethal effect (Fig. 1e) demonstrating that CRISPRi mediates isoform-specific phenotypes. We further validated these observations across the larger panel of 55 pan-essential transcripts. We found that only sgRNAs targeting high expressing pan-essential transcripts had the expected anti-proliferation effect while sgRNAs targeting the low expressing transcripts had no effect on proliferation (Fig. 1f and Additional file 1: Fig. S1A-E). Taken together, our results demonstrate CRISPRi as a reliable tool for inhibiting the expression from specific promoters.

### CRISPRi screen of gastric cancer-associated isoforms identifies isoform-specific dependencies

**Fig. 2:**
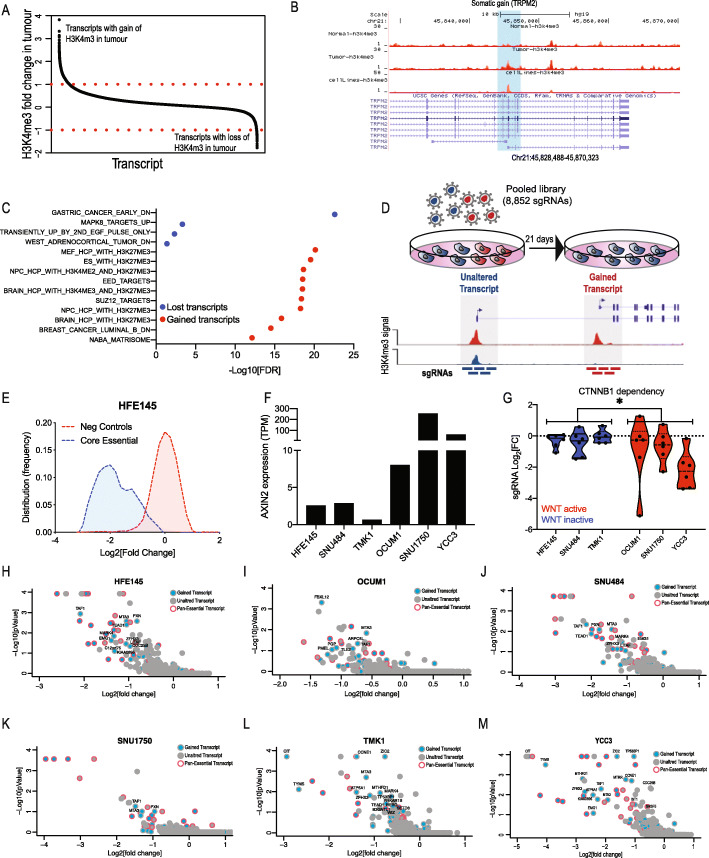
CRISPRi screen identifies GC essential transcript isoforms. **a** Transcripts with gain or loss (compared to adjacent normal) of H3K4me3 signal in GC. Fold change is calculated by comparing H3K4me3 signals in tumour and adjacent normal tissue. H3K4me3 signals are an average of 17 tumour or normal signals from [10]. **b** Example H3K4me3 profiles of a gained GC transcript, *TRPM2*. **c** GSEA showing the top-scoring enriched pathways of transcripts with gain or loss of H3K4me3. **d** Scheme describing the isoform-specific CRISPRi screen. A library containing 8852 sgRNAs  targeting 820 GC gained transcripts was used to identify transcripts that are essential for the proliferation of normal gastric (HFE145) or 5 GC cell lines. **e** Distribution of sgRNAs targeting negative controls or core essential genes. **f** Expression levels of *AXIN2*, a WNT target gene, in GC cell lines. **g** Violin plot showing *CTNNB1* dependency in GC cell lines. Dots represent individual *CTNNB1* targeting sgRNAs. pValue between WNT-active and WNT-inactive cell lines was calculated using two-tailed unpaired *t* test. (*p ≤ 0.05). **h**–**m** MAGeCK analysis [22] was used to identify transcript isoforms that are essential in a normal gastric cell line (HFE145) or 5 GC cell lines. GC gained transcripts are in blue, and unaltered transcripts are in grey. Pan-essential genes (genes that score as essential in ≥ 90% of cell lines in DepMap) are indicated with a red border

Using nano-chip-seq, we have previously measured the abundance of H3K4me3, a histone mark that is present in transcriptionally active promoters, in tumour and adjacent normal tissue from 17 GC patients [10]. By comparing H3K4me3 abundance in tumour and normal pairs from these patients, we identified a set of transcript isoforms that are gained or lost in GC (Fig. 2a and Additional file 2: Table S3). As an example, we show a representative profile of *TRPM2*, a gained GC-associated isoform (Fig. 2b). In both GC patient tumours and cultured cell lines, gain of promoter activity in the *TRPM2* gene leads to the expression of a cancer-specific isoform.

Using gene set enrichment analysis (GSEA) [23], we assessed the pathways enriched in these GC gained or lost isoforms and found that upregulated GC-associated isoforms are enriched with genes whose promoters are regulated during embryonic development (Fig. 2c) consistent with the fact that we identified these active promoters by profiling promoter regulation. GC isoforms that are lost are enriched with known downregulated GC genes (Fig. 2c). These observations suggest that transcript isoforms that are gained or lost in GC drive the expression of functional transcript isoforms.

To assess the functional role of these GC-associated transcript isoforms, we performed a systematic pooled CRISPRi proliferation screen. We selected GC-associated isoforms for screening based on the following criteria: (a) GC-specific promoters with the deregulation of H3K4me3 in ≥ 2 samples (normal vs cancer); (b) RNA-Seq in primary GC shows high isoform expression (FPKM ≥ 5). Based on these criteria, we selected 820 isoforms (Additional file 2: Table S3) and designed a pooled CRISPRi sgRNA library targeting the promoters of these gained/lost isoforms (6 sgRNAs/transcript, Additional file 2: Table S2). For each of these transcripts, we also included sgRNAs targeting an unaltered isoform (Additional file 2: Table S2). Although in many cases, the unaltered transcript is not expressed in these cells (Additional file 2: Table S3), this control enables to determine the specificity of this approach. sgRNAs were designed by identifying the promoter sequence using CAGE-Seq, and the sgRNAs were selected to be ± 150 bp from the TSS. Since some of these regions are highly AT or GC rich, for some of these promoters, only a limited number of sgRNAs are available. To ensure sgRNA specificity, we selected sgRNAs that only aligned once to the human genome. In addition, as positive controls, we included 612 sgRNAs targeting 102 known core cell essential genes [24, 25] and 1064 negative control sgRNAs. Following library transduction, cells were propagated for 21 days followed by DNA extraction and quantification of sgRNA abundance (Fig. 2d). Negative control sgRNAs had no effect, and as expected, suppression of core essential genes had a dramatic anti-proliferation effect (Fig. 2e and Additional file 1: Fig. S2A-E). Furthermore, known oncogenes showed expected dependencies. For example, the WNT/β-catenin pathway is deregulated in 3 of these 6 cell lines, consistent with previous observations [26, 27] and as measured by *AXIN2* expression (Fig. 2f). As expected, β-catenin (*CTNNB1*) targeting sgRNAs had an anti-proliferation effect only in WNT/β-catenin active cell lines (Fig. 2g). To identify GC essential isoforms, we used MAGeCK analysis [22] to calculate isoform dependency scores in 5 GC cell lines (SNU1750, TMK1, SNU484, OCUM1 and YCC3) and one normal immortalised gastric cell line (HFE145) (Additional file 2: Table S4). As statistically significant isoform dependencies, we considered isoforms with an FDR ≤ 0.1. As expected, the majority of scoring transcripts are pan-essential genes (Fig. 2h–m) and are not likely to be directly involved in GC.

**Fig. 3:**
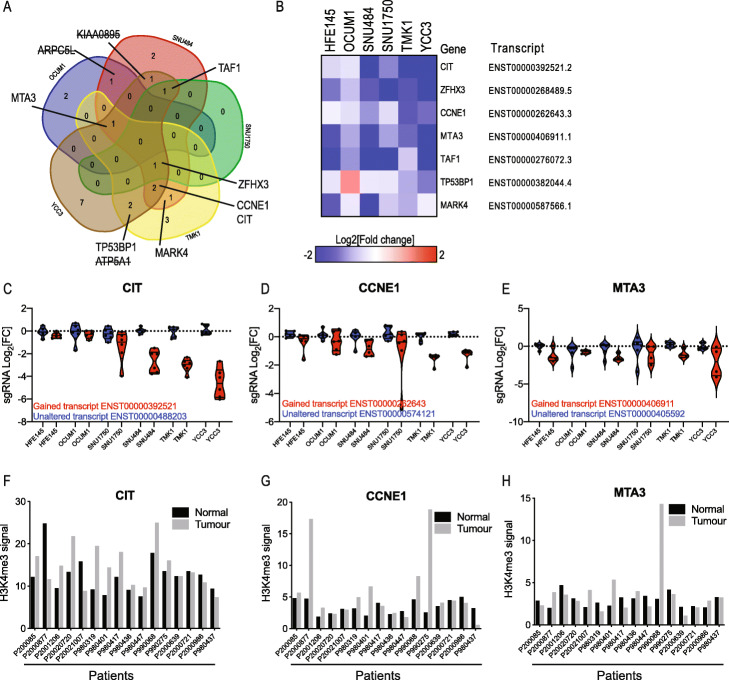
CRISPRi screen identifies GC-specific transcript isoforms. **a** Transcripts with an FDR ≤ 0.1 as determined by MAGeCK analysis were considered significant. Venn diagram showing the transcripts that score in multiple GC cell lines (not including pan-essential genes). Crossed genes exhibit a bidirectional promoter that is shared with a cell-essential gene (Additional file S3). **b** Heatmap showing GC-essential isoform dependencies. HFE145 is a normal gastric cell line. **c**–**e** Violin plots showing sgRNAs targeting *CIT*, *CCNE1* and *MTA3*. Dots represent individual targeting sgRNAs. **f**–**h** H3K4me3 signals in promoter regions of *CIT*, *CCNE1* and MTA3 from GC patient tumour and adjacent normal tissue showing that these essential transcripts are gained in most GC patient samples

To identify GC-gained transcripts that are also essential in multiple GC cell lines, we eliminated pan-essential transcripts and compared transcript dependencies among 5 GC cell lines (Fig. 3a). We found 24 transcripts that were essential in at least 1 cell line; 10 of these transcripts were essential in ≥ 2 cell lines (Fig. 3a). We have previously shown that bidirectional promoters are a major source of off-target effects in CRISPRi screens [17]. Specifically, sgRNAs directed to a particular TSS will also target any TSS located within 1000–2000 bp from that sgRNA. We found that 5 transcripts are located within 2000 bp from another TSS (Additional file 1: Fig. S3A). Three of these 5 transcripts (*ATP5A1*, *ARPC5L* and *KIAA0895*) are located in close proximity to a pan-essential gene and most likely scored due to off-target inhibition of the cell-essential gene (Additional file 1: Fig. S3B). Our analysis revealed 7 transcripts that show a gain of H3K4me3 in GC and are essential for GC proliferation (Fig. 3b). To prioritise these transcripts, we compared GC dependencies to a non-transformed gastric cell line (HFE145) (Fig. 3b). We validated three transcript isoforms which showed greater dependency in GC than in normal immortalised cells including *CIT*, *CCNE1* and *MTA3*. The proliferation phenotype we observed following the introduction of sgRNAs targeting these GC-gained transcripts was isoform-specific. We found that sgRNAs targeting a promoter from the same gene that drives the expression of a transcript that is unaltered, and many times not expressed, had no effect on proliferation (Fig. 3c–e). In consonance, with these observations, H3K4me3 abundance in the promoters of these isoforms was higher in GC tumours compared to adjacent normal tissue (Fig. 3f–h). Thus, we have identified two types of transcripts: (a) transcripts that are not expressed in normal conditions and are gained in cancer (e.g. *CIT*, *CCNE1*) and (b) transcripts that gain an additional cancer-associated transcript (e.g. *MTA3*, *ZFHX3*).

### Validation of *CIT*, *CCNE1* and MTA3 as isoform-specific GC dependencies

**Fig. 4:**
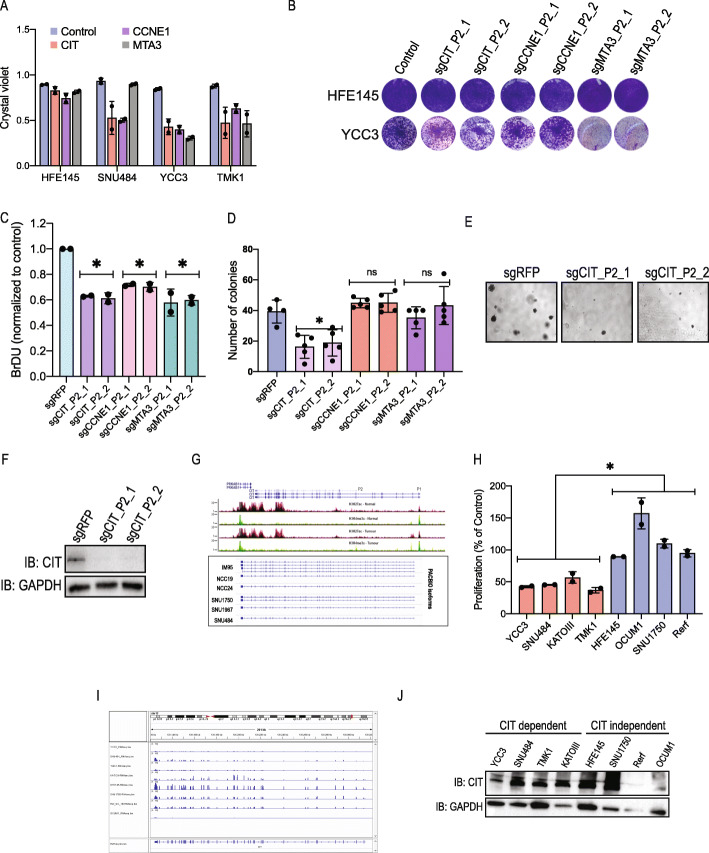
Validation of GC-essential isoforms. **a** Crystal violet proliferation assay following CRISPRi-mediated suppression with sgRNAs targeting CIT, CCNE1 and MTA3. Data is shown as mean ± SD, *n* = 2; each dot is a different sgRNA. **b** Images from crystal violet proliferation assay of HFE145 (normal gastric cell line) or YCC3 (GC cell line) 7 days post-infection with sgRNAs targeting *CIT*, *CCNE1* or *MTA3*. **c** BrDU proliferation assay. 7 DPI with the indicated sgRNAs. Data is shown as mean ± SD, *n* = 2; each dot is a biological replicate of the indicated sgRNA. pValue is calculated using two-tailed unpaired *t* test. (*p ≤ 0.05). **d** Anchorage-independent growth of YCC3 cells following CRISPRi-mediated suppression of the indicated genes. Following sgRNA infection, 50,000 cells were plated on a semisolid surface. A number of colonies were quantified by counting 5 different images for each sgRNA. Data is shown as mean ± SD; each dot is a different image. pValue is calculated using two-tailed unpaired *t* test. (*p ≤ 0.05). **e** Images of YCC3 colonies in control cells or following suppression of CIT expression. **f** WB showing the CIT expression in YCC3 cells following CRISPRi-mediated suppression. **g** H3K4me3 and H3K27Ac at the *CIT* promoter in GC tumours and adjacent normal tissue. Bottom panel shows the *CIT* PacBio sequencing in GC cell lines. **h** The indicated GC cell lines were infected with control or CIT-targeting sgRNAs (two sgRNAs). Seven days post-infection, cell proliferation was assessed using a crystal violet staining assay. The results are plotted as an average ± SD of two sgRNAs. Each dot represents a different sgRNA. pValue is calculated using two-tailed unpaired *t* test. (*p ≤ 0.05). **i** *CIT* RNA-Seq reads in GC cell lines. **j** CIT protein levels in GC cell lines

To validate these observations, we cloned individual sgRNAs targeting these GC-gained transcripts (Additional file 2: Table S7). Following sgRNA infection, we used a crystal violet proliferation assay as a readout and found that all the sgRNAs that scored in our screen also inhibited proliferation as individual sgRNAs (Fig. 4a, b). Consistent with these observations, all sgRNAs that showed a proliferation phenotype inhibited target expression (Additional file 1: Fig. S4A-C). Since out of these transcripts only *MTA3* expresses two unique isoforms (Additional file 2: Table S3), we confirmed that the *MTA3* sgRNAs inhibit only the targeted isoform (Additional file 1: Fig. S4C). Similar to what we observed with *TRPM2* (Fig. 2b), the non-essential transcript had very low levels of expression in these cells. We further observed a similar anti-proliferation effect using a BrDU assay (Fig. 4c) demonstrating the reliability of these observations. As an additional approach, we used an anchorage-independent growth assay in YCC3 cells. We found that suppression of *CIT* had a dramatic effect on anchorage-independent growth while *CCNE1* or *MTA3* did not show a decrease in colony formation (Fig. 4d–f).

Of the two *CIT* promoters, only P1 drives the expression of a transcript that is gained and essential in GC (Fig. 4g). We validated these observations using another histone mark (H3K27ac) and by isoform-specific PacBio long-read mRNA sequencing (Fig. 4g). To further validate *CIT* dependency, we used a larger panel of GC cell lines (Fig. 4h). Although H3K4me3 is gained in most of these cell lines (Additional file 2: Table S3), only four of these eight cell lines were *CIT*-dependent (Fig. 4h). *CIT* mRNA and protein were expressed in all *CIT*-dependent cell lines (Fig. 4i, j). Two of the *CIT*-independent cell lines (Rerf, OCUM1) had very low CIT expression and no detectable CIT protein (Fig. 4i, j) suggesting that in these cell lines, H3K4me3 signals did not result in *CIT* expression. Surprisingly, we found that although *CIT* mRNA and protein levels were high in HFE145 and SNU1750 (Fig. 4i, j), these cells were not sensitive to suppression of *CIT* (Fig. 4h). This type of de novo resistance is found for many known oncogene dependencies and is typically associated with activation of alternative pathways.

**Fig. 5:**
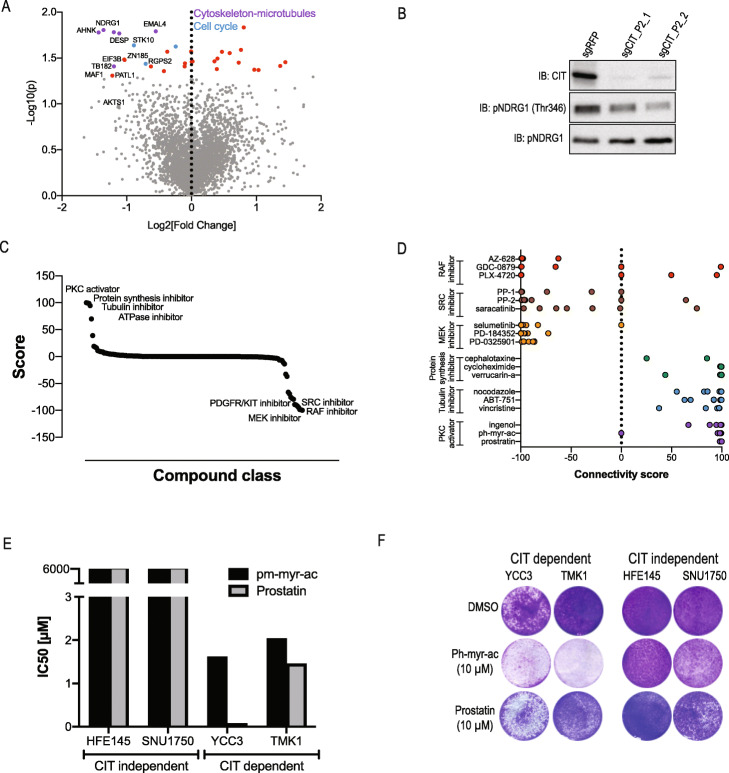
CIT regulates the cytoskeletal and microtubule network in GC cell lines. **a** Phosphoproteins detected in YCC3 cells following CRISPRi-mediated suppression of CIT expression (results are shown as an average of two CIT sgRNAs in duplicate). **b** Validation of NDRG1 phosphorylation regulated by CIT in YCC3 cells. Seven DPI with CIT sgRNAs WB analysis was used to quantify the protein levels of CIT, NDRG1 and phspho-NDRG1 (Thr346). **c** Compound classes that score as associated with the CIT expression signature using the L1000 platform [29]. **d** Individual compounds within drug classes that score as associated with CIT signature. **e** CIT-dependent or CIT-independent cell lines were treated with PKC activators, and proliferation was assessed after 7 days using a crystal violet staining assay. **f** Images of crystal violet staining following treatment with PKC activators

CIT is a serine/threonine kinase that is required for localisation of the kinesin KIF14 and plays a role in cytokinesis [28]. Consistent with these reports, *CIT* and *KIF14* CRISPRko dependency profiles [20] are highly correlated (Additional file 1: Fig. S5A) suggesting a similar function. To gain insights into the cellular processes regulated by CIT in GC, we used mass spectrometry to quantify phosphorylated proteins following *CIT* knockdown (Additional file 2: Table S5). Among the 15 proteins with a significant phosphorylation reduction (*p* < 0.05), 6 (40%) are known cytoskeleton regulators (Fig. 5a).

Phosphorylation of NDRG1, a regulator of the cytoskeleton that has been implicated in cancer migration and invasion [30, 31], was dramatically downregulated following the suppression of CIT expression. We further validated these findings using a phospho-NDRG1 antibody (Fig. 5b). These observations identify CIT as a regulator of the cytoskeleton and suggest CIT inhibitors as a strategy to inhibit cancers with a gain of *CIT* expression.

To identify CIT inhibitors, we queried the L1000 drug repurposing database [29]. Specifically, following *CIT* suppression in YCC3 cells, we used RNA-Seq to measure the changes in the gene expression (Additional file 1: Fig. S5B and Additional file 2: Table S6). By selecting the top 100 up- and downregulated genes, we generated a CIT signature that was used to query the L1000 expression signature database [29]. Consistent with our proteomic analysis, we found that the CIT and “tubulin inhibitor” signatures were highly correlated (Fig. 5c, d). We also found a strong connection between CIT and the “PKC activator” signature. We validated these findings, by treating CIT-dependent and CIT-independent cell lines with two PKC activators (Fig. 5e, f). PKC activators had a dramatic inhibitory response on CIT-sensitive GC cell lines. However, PKC activators did not inhibit CIT targets such as NDRG1 phosphorylation (Additional file 1: Fig. S5C-F). These results further confirm CIT as a cytoskeleton regulator and demonstrate PKC activators as drug mimetics of CIT.

### Promoter specific CRISPRi screens identify isoform-specific functions for the tumour suppressor *ZFHX3*

**Fig. 6:**
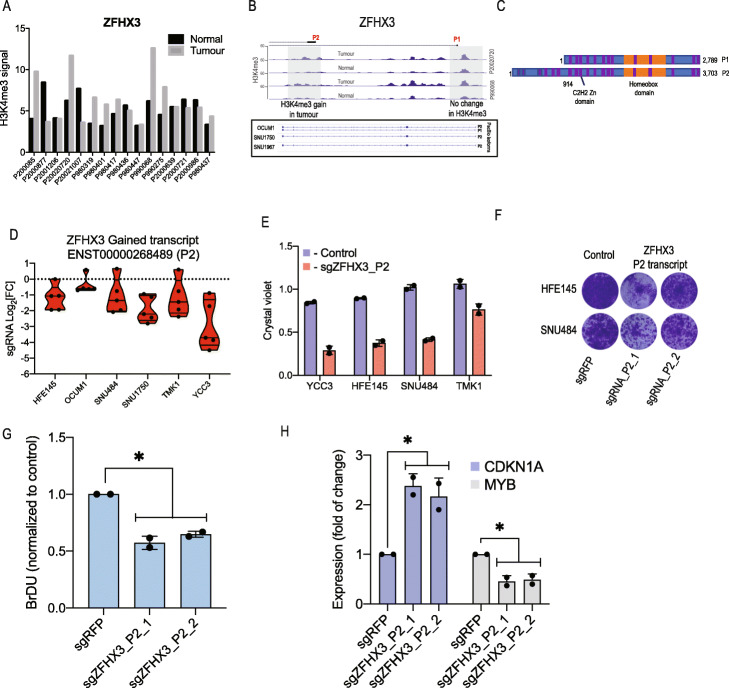
Identification of genes that express alternative transcripts with opposing functions. **a** H3K4me3 signals in the promoter of ZFHX3 in tumour or adjacent normal tissue. Fifty per cent of GC patients show a gain of H3K4me3 at the *ZFHX3* promoter. Each bar represents a tumour or normal sample from the indicated GC patient tissue obtained using nano-chip-seq [10]. **b** Profiles of H3K4me3 signals in two patient samples with a high signal, showing a tumour-specific gain of transcript P2. Bottom panel shows the PacBio reads of ZFHX3 from GC cell lines. **c** Schematic describing the protein generated from P1 or P2 promoters of ZFHX3. **d** Violin plot showing the proliferation changes from the CRISPRi screen induced by sgRNAs targeting transcript P2 of ZFHX3. Each dot represents a different sgRNA. **e** Proliferation changes following CRISPRi-mediated targeting of ZFHX3 transcript P2, measured using crystal violet staining assay in GC cell lines 7 days post-infection. The results are plotted as an average ± SD of two sgRNAs, *n* = 2. Each dot represents a different sgRNA. **f** Representative images from **e**. **g** Proliferation of YCC3 cells measured 7 DPI with sgRNAs targeting ZFHX3 transcript P2 using BrDU proliferation assay. The results are plotted as an average ± SD, *n* = 2. Each dot represents an independent experiment. pValue is calculated using *t* test (*p* ≤ 0.05). **h** qPCR of ZFHX3 target genes following the suppression of ZFHX3 transcript P2. RNA extracted 5 DPI with ZFHX3 sgRNAs was used for qPCR of *CDKN1A* or *MYB*. The results are plotted as an average ± SD, *n* = 2. Each dot represents an independent experiment. pValue is calculated using *t* test (*p* ≤ 0.05)

*ZFHX3* is known as a tumour-suppressor gene (TSG) that is frequently mutated in various cancer types [32, 33]. Opposite to what we expected from a TSG, we found that 50% (8/16) of GC tumours had upregulated H3K4me3 on the *ZFHX3* promoter (Fig. 6a). *ZFHX3* has two isoforms that are expressed from two promoters (Fig. 6b); however, only P2 showed GC-specific gain of H3K4me3 (Fig. 6b). Consistent with this observation, PacBio sequencing identified the P2 transcript in cancer cell lines (Fig. 6b). The two *ZFHX3* isoforms encode a similar catalytic protein; however, the P2 transcript has additional 914aa at its N terminus (Fig. 6c).

In consonance with these observations, we found that CRISPRi-mediated suppression of *ZFHX3* isoform P2 inhibited proliferation in all 6 cell lines we tested (Fig. 6d). We validated these findings using individual *ZFHX3* isoform P2 targeting sgRNAs (Fig. 6e, f and Additional file 1: Fig. S6). As a complementary assay, we validated that the *ZFHX3* isoform P2 is required for proliferation using BrDU staining in YCC3 cells (Fig. 6g).

Since *ZFHX3* is a transcription factor to evaluate the mechanism of *ZFHX3* isoform P2, we measured how suppression of this isoform affects the expression of known *ZFHX3* target genes. Previous reports have shown that the tumour suppressor isoform of ZFHX3 (isoform P1) upregulates the expression of the tumour suppressor *CDKN1A* and downregulates the expression of the oncogene *MYB* [32, 34]. Suppression of *ZFHX3* isoform P2 resulted in the opposite expression pattern (Fig. 6h) suggesting a negative loop feedback mechanism.

### In vivo validation of *CIT* and *ZFHX3*

**Fig. 7:**
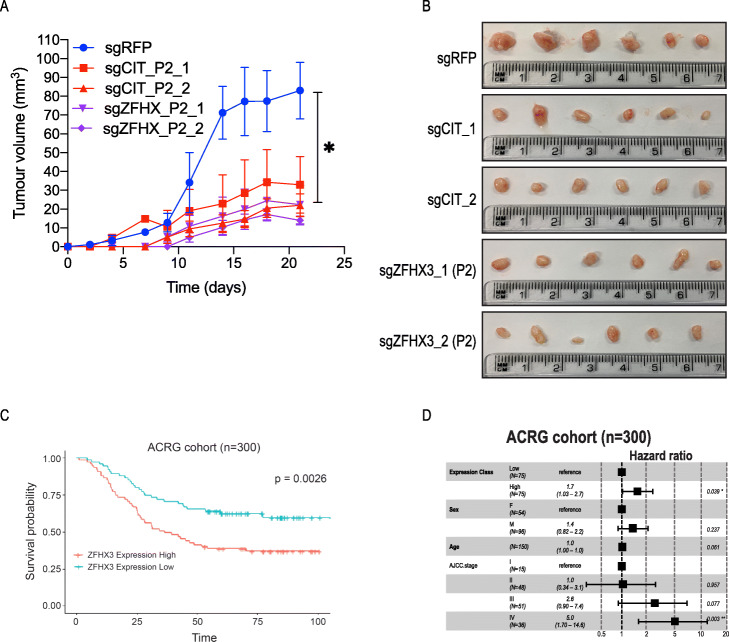
High expression of ZFHX3 predicts the worse outcome of GC patients. **a** Four DPI of YCC3 cells with the indicated sgRNAs 2e6/site were injected to three sites of immune-deficient mice. The results are shown as average ± SD, *n* = 6. pValue is calculated by comparing the control to each of the sgRNA-treated samples using the *t* test (*p* ≤ 0.05). **b** Tumours extracted from xenograft. **c** Kaplan-Meier survival plot and **d** subclass by patient type using the ACRG patient cohort

To further explore the relevance of these findings in GC patient populations, we assessed *CIT* and *ZFHX3* dependency in mouse xenografts. YCC3 cells expressing *CIT* or *ZFHX3* sgRNAs were injected subcutaneously into immune-deficient mice. Similar to what we observed in cultured cell lines, CRISPR-mediated suppression of *CIT* or *ZFHX3* led to the reduction in expression of the target (Additional file 1: Fig. S7A,B) and tumour size (Fig. 7a, b).

Based on these results, we tested if the expression of these transcripts is predictive of GC patient outcomes used two patient cohorts (TCGA and ACRG). Although CIT expression was not correlated with patient outcomes (Additional file 1: Fig. S7C,D), we found a strong correlation between *ZFHX3* expression and patient survival. In accordance with our model and consistent with the role of the P2 transcript as an oncogene, we found that that high expression of *ZFHX3* was significantly correlated with patient poor survival in both the ACRG (Fig. 7c, d) and the TCGA (Additional file 1: Fig. S7E,F) cohorts. Taken together, we conclude that these two *ZFHX3* isoforms encode proteins with opposing functions which may be a general mechanism for the regulation of protein function.

## Discussion

Promoter usage is a major driver of transcript isoform expression. Methods to measure isoform-specific expression such as CAGE-Seq and histone ChIP-Seq are becoming more widely used and leading to the discovery of isoform-specific expression that is tissue- and disease-specific [8–10]. It is becoming clear that isoform expression has important roles in regulating biological processes; yet, we lack tools for systematic evaluation of promoter-driven transcript isoforms.

RNAi could be targeted to isoform-specific sequences and could be used for isoform-specific studies. However, seed-driven off-target effects complicate the interpretation of RNAi-driven data [35]. Traditional CRISPRko approaches are typically aimed at targeting the first few exons of a gene and are not be able to distinguish between different promoters [25]. Furthermore, since in many cases isoforms share large portions of their sequence, it is difficult to generate isoform-specific CRISPRko sgRNAs. Our work demonstrates the ability of CRISPRi to target alternative promoters that drive the expression of functional transcript isoforms. CRISPRi works only at a defined window from the TSS and as such could be used for isoform-specific suppression.

In the present study, looking at isoform-specific GC transcripts, we found the serine/threonine kinase CIT as a dependency in cells with gain of H3K4me3 signal on a defined promoter. In these experiments, we also identified two cell lines (OCUM1 and Rerf) which although had the H3K4me3 signal did not show any *CIT* mRNA or protein. These observations suggest a possible post-transcriptional inhibitory mechanism and emphasis that histone mark changes do not always lead to a gene expression outcome. Importantly, these findings demonstrate that *CIT* is only required in a specific context and suggest that CIT inhibitors will not have a general toxic effect. CIT has previously been reported as a potential target in breast [36], colon [37] and brain [38] cancers. Here, we show that GC cell lines with expression gain of a specific CIT isoform are highly sensitive to CRISPRi-mediated CIT suppression. Furthermore, our results using proteomics and expression profiling demonstrate CIT as a critical regulator of the cytoskeleton network and suggest CIT expression as a cancer treatment biomarker. CIT did not score as a specific dependency in DepMap [20] using CRISPRko across a large panel of cancer cell lines. This is most likely due to the fact that CRISPRko sgRNAs do not take isoforms into consideration, and those miss these isoform-specific signals. Using the L1000 drug repurposing hub, we identified and validated PKC activators as small molecules that mimic the effect of genetic suppression of *CIT* expression. Although further studies are needed into how PKC activators modulate CIT activity, these results suggest a path towards the development of potent CIT inhibitors.

Our work shows that in some cases, transcripts from the same gene could have opposite functions. Other such known examples include BCL-xL and BCL-xS, two *BCL2L1* transcripts with opposing functions [12]. Although we currently do not know the extent of this phenomena, this should be considered when designing genetic screens. Most current CRISPR libraries target the entire gene and do not take into account transcript variants. Our results demonstrate that ZFHX3 isoform P2 functions as an oncogene. This may represent an opportunity for differential mutational control during cancer progression. Further analysis of cancer mutations focused on the location of mutations in relation to isoform expression may shed a new light on this type of genes.

We show that transcript usage could explain some inconsistencies between functional and structural genomics. Furthermore, these results imply that simple expression patterns may be misleading when trying to interpret the function of a gene. Although this study was focused on identifying alternative promoters that are essential for GC pathogenesis the same approach could be used as a general strategy to functionally classify transcript isoforms.

## Methods

### Cell lines and media

Cell lines used in this study were obtained from Japan Health Science Research Resource Bank (KATOIII, OCUM1, Rerf) and Korean Cell Line Bank (SNU484, SNU1750); HFE145 was gifted by Dr. Hassan Ashktorab, Howard University; and YCC3 was gifted by Yonsei Cancer Centre, South Korea and maintained in a 37 °C incubator, 5% CO_2_. All cell lines were propagated in media containing 10% FBS and 1% NEAA in KatoIII - RPMI, OCUM1 - DMEM with 0.5 mM Na-Pyruvate, HFE145 - DMEM, SNU484 - RPMI, YCC3 - MEM, SNU1750 - RPMI and Rerf - RPMI. Cell lines (besides OCUM1) were authenticated by STR genotyping performed at the Cancer Science Institute of Singapore. OCUM1 cells were authenticated by spotting live cells onto an ATCC CLA card and sending to the vendor for analysis.

### Generation of pooled sgRNA library

sgRNA spacers (Additional file 2: Table S2) were designed to target altered and unaltered gastric cancer promoters. For each promoter, we designed 6 sgRNAs. sgRNA pooled libraries were made as previously described [17]. Briefly, oligo pools were purchased from custom arrays (WA, USA) containing sgRNAs and flanking PCR handles with BsmBI cutting sites (AGGCACTTGCTCGTACGACGCGTCTCACACCG[20ntsgRNA]GTTTCGAGACGTTAAGGTGCCGGGCCCACAT). Following PCR amplification, golden gate cloning was used to insert this library into the BsmBI sites of lentiGuide-Puro vector (Addgene catalogue no. #52963). Following electroporation of this library to DH5α bacterial cells ensuring at least 1000× representation, maxi prep (Qiagene) was used to extract the DNA.

### CRISPRi screen

CRISPRi pooled proliferation screens were done as previously described [17]. Briefly, GC cells stably expressing dCas9-KRAB (Addgene: 89567) were infected at a MOI of 0.3 at a multiplicity of 1000 cells/sgRNAs. Twenty-four hours post-infection, infected cells were selected using puromycin (2 μg/ml). To ensure sgRNA and dCas9-KRAB, expression cells were maintained with puromycin and blastocidine throughout the screen. Twenty-one days post-infection, genomic DNA was extracted using NucleoSpin Blood XL (Clontech), and the sgRNA-Seq was amplified as previously described. sgRNA was quantified using HiSeq Illumina sequencing. Deconvolution of sgRNA read counts was done using the poolQ algorithm with default settings (https://portals.broadinstitute.org/gpp/public/software/poolq).

### MAGeCK analysis

MAGeCK algorithm [22] was used for defining gene scores and for identifying gene essentiality significance. Default settings were used. Reads from replicate samples at day 21 were compared to reads from the DNA pool. Transcripts with an FDR ≤ 0.1 were considered as hits.

### RNA isolation and quantitative real-time PCR

Total RNA was isolated using TRIzol (Sigma Aldrich) from mouse tumours and cell lines, according to the manufacturer’s instructions. One microgram of total RNA was reverse transcribed to cDNA using the Maxima Reverse Transcriptase (Thermo Fisher) in 20 μl reaction with random hexamers and dNTP. Expression levels of genes were quantified using qRT-PCR using the QuantStudio™5 Real-Time PCR system (Thermo Fisher). qRT-PCR was performed using the indicated qPCR primers (Additional file 2: Table S7). GAPDH was used for normalisation. The qRT-PCR conditions were denaturation at 95 °C for 5 min followed by 40 cycles of amplification at 95 °C for 15 s and 60 °C for 20 s. The comparative cycle threshold (delta Ct) method was used to analyse the gene expression levels.

### BrDU cell proliferation assay

The BrDU Cell Proliferation Assay Kit (Cat. No. #6813, Cell Signaling Technology) was used to investigate proliferation upon different gene knockdowns. A total of 5000 cells were seeded in 100 μl of media on a 96-well plate; following virus infection, cells were incubated at 37 °C for 5 days. BrDU was then added to the cells which were incubated for 6 h at 37 °C to incorporate BrDU during S phase. Subsequent procedure was performed according to the manufacturer’s instructions. The BrDU incorporation was measured at 450 nm with the CLARIOstar microplate reader (BMG Labtech) using the MARS Data Analysis software (BMG Labtech).

### Crystal violet proliferation assay

Following sgRNA infection and selection, cells were allowed to propagate for the indicated time. The media were removed, and cells were washed twice in PBS. 10% of formalin in PBS was added and incubated for 20 min at room temperature. Formalin was removed, and 0.5% (w/v) of crystal violet (Sigma # C0775-25G) was added and incubated for 20 min at room temperature. Crystal violet was removed, and plates were thoroughly washed with PBS. For quantification, 10% of acetic acid was added to each well and incubated at room temperature for 30 min. The extracted solution was added to a 96-well plate and quantified by measuring the OD at 590 nm.

### Anchorage-independent growth assay

YCC3 cells (5 × 10^5^) were seeded in 0.3% Noble agar (Sigma, St. Louis) in 6-well plates, three replicates/sample. Bottom agar consisted of cell line media with 0.6% Noble agar. Colony formation was assessed at 3 weeks, and images of each well were taken at a × 5 magnification using an EVOS microscope (Thermo).

### Western blot

Western blot was done using BioRad pre-casted gels and a trans-turbo transfer machine (BioRad). The following are the antibodies used in this study: CIT antibody (BD Biosciences # 611376), NDRG1 (Cell Signaling # 9485), phospho-NDRG1 (Cell Signaling # 5482) and GAPDH (Sana Cruz # *SC32233*).

### RNA-Seq following CRISPR-mediated gene suppression

Transcript abundance was estimated from the RNA-Seq data against the human genome reference GRCh37.p13, using transcript annotations taken from the GENCODE project v19 annotation. Transcript sequences were extracted for this annotation using RSEM v1.3.2 [39], then transcript quantification performed using Salmon v0.14.1 [21] and abundance estimates reported in transcripts per million (TPM).

### Phospho-proteomic mass spec

Cells were lysed with chilled 4% (w/v) sodium deoxycholate to a final volume of 270 μl. Approximately 500 μg of protein lysate was reduced and alkylated before overnight tryptic enzymatic digestion, as previously described [40]. Briefly, phosphopeptides were enriched with titanium dioxide beads and desalted prior to liquid chromatography-tandem mass spectrometry (LC-MS/MS) analysis with a QExactive mass spectrometer (Thermo Scientific) in the Monash Proteomics and Metabolomics Facility [41]. The instrument was operated in the data-dependent acquisition mode to automatically switch between full-scan MS and MS/MS acquisition. Data were processed with MaxQuant (v1.6.0.16) with standard parameters for phosphopeptide quantification [40]. Log2 intensities were summarised across all samples in a linear mixed model implemented in the R package Limma [42] for pairwise comparison for each phosphopeptide.

### PacBio sequencing

Ten cell lines were selected for PacBio long-read RNA sequencing. Each cell line was sequenced using four SMRT wells. For each sample, we used the CCS module of IsoSeq3 program (https://github.com/PacificBiosciences/IsoSeq3) to generate circular consensus sequence (CCS) reads from the sub-reads generated from the sequencing run. The reads are classified into full-length non-chimeric (FLNC) and non-FLNC reads. Following this, the reads that are identified as FLNC are considered for de novo clustering of reads using the cluster module of IsoSeq3 to identify unique isoforms. All isoforms are mapped to the human genome (version hg38) using GMAP [43], and only high-quality isoforms (supported by at least two full-length non-chimeric reads) are considered for further analysis. Further quality control and isoform annotations were performed using SQANTI2 (https://github.com/Magdoll/SQANTI2).

### Mouse xenografts

All animal studies were approved by the Monash University Animal Ethics Committee (AEC – approval number 2020-24197-49078). YCC3 stably expressing KRAB-dCas9 were infected with lentiviruses containing the indicated sgRNAs. Following puromycin selection (3 DPI), 2 million cells/site and 3 sites/mouse were subcutaneously injected into 5-week-old female NSG mice (a kind gift from Professor Gail Risbridger and A/Prof. Renea Taylor, Monash University) under isofluorane anaesthesia. Tumour growth was continuously monitored for 4 weeks. Tumours were measured using digital vernier calliper every 48 h, beginning 3 days after injection, and tumour volume was calculated using the formula length (mm) × width (mm) × height (mm) and expressed in mm^3^.

## Supplementary Information

**Additional file 1:** **Figure S1.** CRISPRi identifies expressed pan-essential transcripts. Distribution of sgRNAs targeting high- or low-expressed pan cell-essential transcripts. **Figure S2.** CRISPRi screen quality control. Distribution of sgRNAs targeting negative controls or core essential genes. **Figure S3.** Essential transcripts that score due to a bidirectional promoter. Description of transcripts that score due to off-target bi-directional promoters. **Figure S4**. Validation of GC-essential transcripts. qPCR of sgRNAs targeting transcripts that are validated in this study. **Figure S5.** CIT dependency in GC. CIT dependency in DepMap and pNDRG1 levels following treatment with Phorbol 12-myristate 13-acetate or prostratin. **Figure S6**. ZFHX3 isoform P2 expression following CRISPRi-mediated suppression of transcript P2. **Figure S7.** In vivo validation of ZFHX3 and CIT**.** CIT and ZFHX3 levels from tumour xenografts and Kaplan-Meier survival plot of GC patients.

**Additional file 2:** **Table S1.** Transcript specific RNA-Seq (using the Salomon algorithm) in 4 gastric cancer cell lines. **Table S2.** Spacer sequences of sgRNAs used in pooled library. **Table S3.** Gastric cancer specific altered and un-altered transcripts. **Table S4.** Results from CRISPRi screen. Data was generated using the MAGeCK algorithm. **Table S5.** Phospho-proteomic mass spectrometry in YCC3 cells following CRISPRi mediated suppression of CIT expression. **Table S6.** RNA-Seq in YCC3 cells following CRISPRi mediated suppression of CIT expression. **Table S7.** Primers used in this study for qPCR and generation of sgRNAs.

**Additional file 3:** Review history.

## Data Availability

All the datasets supporting the conclusions of this article are available in the tables included in this manuscript.
