# Author Correction: CRISPRi enables isoform-specific loss-of-function screens and identification of gastric cancer-specific isoform dependencies

**DOI:** 10.1186/s13059-021-02314-1

**Published:** 2021-03-25

**Authors:** Rebecca Davies, Ling Liu, Sheng Taotao, Natasha Tuano, Richa Chaturvedi, Kie Kyon Huang, Catherine Itman, Amit Mandoli, Aditi Qamra, Changyuan Hu, David Powell, Roger J. Daly, Patrick Tan, Joseph Rosenbluh

**Affiliations:** 1 Cancer Research Program and Department of Biochemistry and Molecular Biology, Biomedicine Discovery Institute, Monash University, Clayton, VIC 3800 Australia; 2 Program in Cancer and Stem Cell Biology, Duke-NUS Medical School, Singapore, 169857 Singapore; 3 Cancer Science Institute of Singapore, National University of Singapore, Singapore, 117599 Singapore; 4 Cancer Therapeutics and Stratified Oncology, Genome Institute of Singapore, Singapore, 138672 Singapore; 5 SingHealth/Duke-NUS Institute of Precision Medicine, National Heart Centre Singapore, Singapore, 169856 Singapore; 6 Cellular and Molecular Research, National Cancer Centre, Singapore, 169610 Singapore; 7 Singapore Gastric Cancer Consortium, Singapore, 119074 Singapore; 8 Functional Genomics Platform, Monash University, Clayton, VIC 3800 Australia; 9 Monash Bioinformatics Platform, Monash University, Clayton, VIC 3800 Australia

**Correction to: Genome Biol (2021) 22:47**

**https://doi.org/10.1186/s13059-021-02266-6**

Following publication of the original paper [1], the authors reported an error. In Fig. 1c we have mistakenly mislabeled the sgRNAs. The corrected Fig. 1 is given in this correction article.

**Fig. 1:**
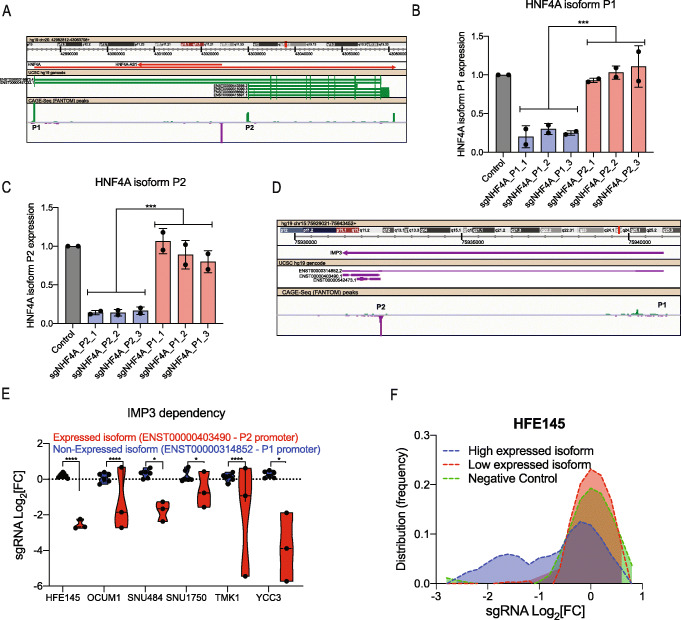
CRISPRi as a tool for inhibition of specific promoter-driven transcript isoforms. **a** Structure of the *HNF4A* gene. Isoforms P1 and P2 are marked. CAGE-Seq peaks from the FANTOM project [19] are shown in the bottom panel. **b** qRT-PCR quantification of *HNF4A* transcript P1 following CRISPRi-mediated suppression of transcript P1 or P2. Data is shown as mean ± SD, *n* = 2. pValue is calculated using two-tailed unpaired *t* test (***p ≤ 0.001). **c** qRT-PCR quantification of *HNF4A* transcript P2 following CRISPRi-mediated suppression of transcript P1 or P2. Data is shown as mean ± SD, *n* = 2. pValue is calculated using two-tailed unpaired *t* test (***p ≤ 0.001). **d** Structure of the *IMP3* gene. Isoforms P1 and P2 are marked. CAGE-Seq peaks from the FANTOM project are shown in the bottom panel. **e** Violin plot showing IMP3 dependency following CRISPRi-mediated suppression of different isoforms in GC cell lines. Dots represent individual sgRNAs targeting the indicated *IMP3* transcript isoform. pValue is calculated using two-tailed unpaired *t* test (****p ≤ 0.0001, *p ≤ 0.05). **f** Distribution of sgRNAs targeting different transcript isoforms of 55 pan cell-essential transcripts. Green, negative control sgRNAs. Purple, sgRNAs targeting the highest expressed (based on RNA-Seq) transcript isoform. Red, sgRNAs targeting the low expressed (based on RNA-Seq) transcript isoform
