# Supplementary material for: CRISPRi enables isoform-specific loss-of-function screens and identification of gastric cancer-specific isoform dependencies
Source: Genome Biol. 2021 Jan 26;22:47. doi: 10.1186/s13059-021-02266-6 (PMC7836456; doi:10.1186/s13059-021-02266-6)
Supplement: Supplementary file 1 — Additional file 1: Figure S1. CRISPRi identifies expressed pan-essential transcripts. Distribution of sgRNAs targeting high- or low-expressed pan cell-essential transcripts. Figure S2. CRISPRi screen quality control. Distribution of sgRNAs targeting negative controls or core essential genes. Figure S3. Essential transcripts that score due to a bidirectional promoter. Description of transcripts that score due to off-target bi-directional promoters. Figure S4. Validation of GC-essential transcripts. qPCR of sgRNAs targeting transcripts that are validated in this study. Figure S5. CIT dependency in GC. CIT dependency in DepMap and pNDRG1 levels following treatment with Phorbol 12-myristate 13-acetate or prostratin. Figure S6. ZFHX3 isoform P2 expression following CRISPRi-mediated suppression of transcript P2. Figure S7. In vivo validation of ZFHX3 and CIT. CIT and ZFHX3 levels from tumour xenografts and Kaplan-Meier survival plot of GC patients. [file 13059_2021_2266_MOESM1_ESM.pdf]

Supplemental Figures legend

Figure S1: CRISPRi identifies expressed pan-essential transcripts. (A-E) Distribution of sgRNAs targeting high- or low-expressed pan cell-essential transcripts.

Figure S2: CRISPRi screen quality control. (A-E) Distribution of sgRNAs targeting negative controls or core essential genes.

Figure S3: Essential transcripts that score due to a bidirectional promoter. (A) All GC-essential transcripts that are expressed from a bidirectional promoter (defined as a second transcript at a distance of <2,000bp). (B) Proliferation scores (CERES scores) from project Achilles for genes exhibiting bidirectional promoters. *ARPC5L*, *ATP5F1A* and *KIAA08595* score due to close proximity to a cell essential gene and are excluded.

Figure S4: Validation of GC-essential transcripts. Quantitative RT-PCR in YCC3 cells 5 days post infection with sgRNAs targeting (A) CIT (B) CCNE1 (C) MTA3. For MTA3 sgRNAs targeting isoform P1 and P2 are shown. CIT and CCNE1 only express one isoform. Results are plotted as an average ±SD n = 2. pValue calculated using two tailed unpaired t-test. (*, $p \le 0.05$).

Figure S5: CIT dependency in GC. (A) CIT and KIF14 CERES scores from project Achilles plotted against each other. (B) RNA-Seq following suppression of CIT expression in YCC3 cells. (C,D) pNDRG1 levels following 24h treatment with the

indicated concentration of Phorbol 12-myristate 13-acetate (E,F) pNDRG1 levels following 24h treatment with the indicated concentration of prostratin.

Figure S6: ZFHX3 isoform P2 expression following CRISPRi-mediated suppression of transcript P2. Results are plotted as an average ±SD, n = 2. pValue calculated using two tailed unpaired t-test. (*, $p \leq 0.05$).

Figure S7: In vivo validation of ZFHX3 and CIT. (A) CIT protein levels from tumour xenografts. (B) ZFHX3 mRNA levels from tumour xenografts. Results are plotted as an average ±SD. Each dot represents a different xenograft tumour. pValue calculated using two tailed unpaired t-test. (*, $p \leq 0.05$). (C) Kaplan-Meier survival plot of GC patients expressing CIT using the ACRG cohort or the (D) TCGA cohort. (E) Kaplan-Meier survival plot and (D) subclass by patient type of ZFHX3 expression using the TCGA patient cohort.

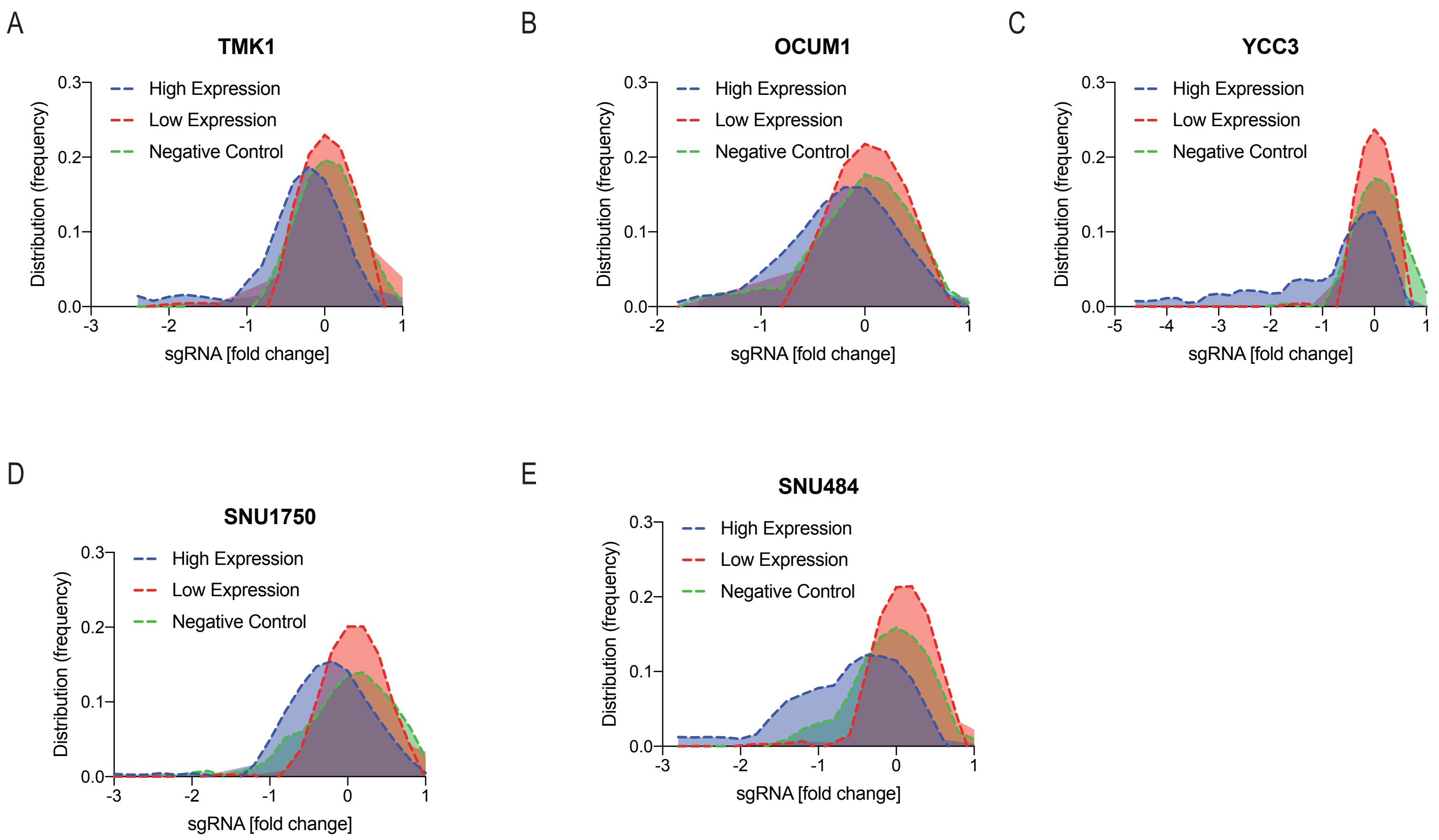
A
TMK1
B
OCUM1
C
YCC3
D
SNU1750
E
SNU484
High Expression
Low Expression
Negative Control
Distribution (frequency)
sgRNA [fold change]
0.0
0.1
0.2
0.3
-5
-4
-3
-2
-1
0
1

Figure S1

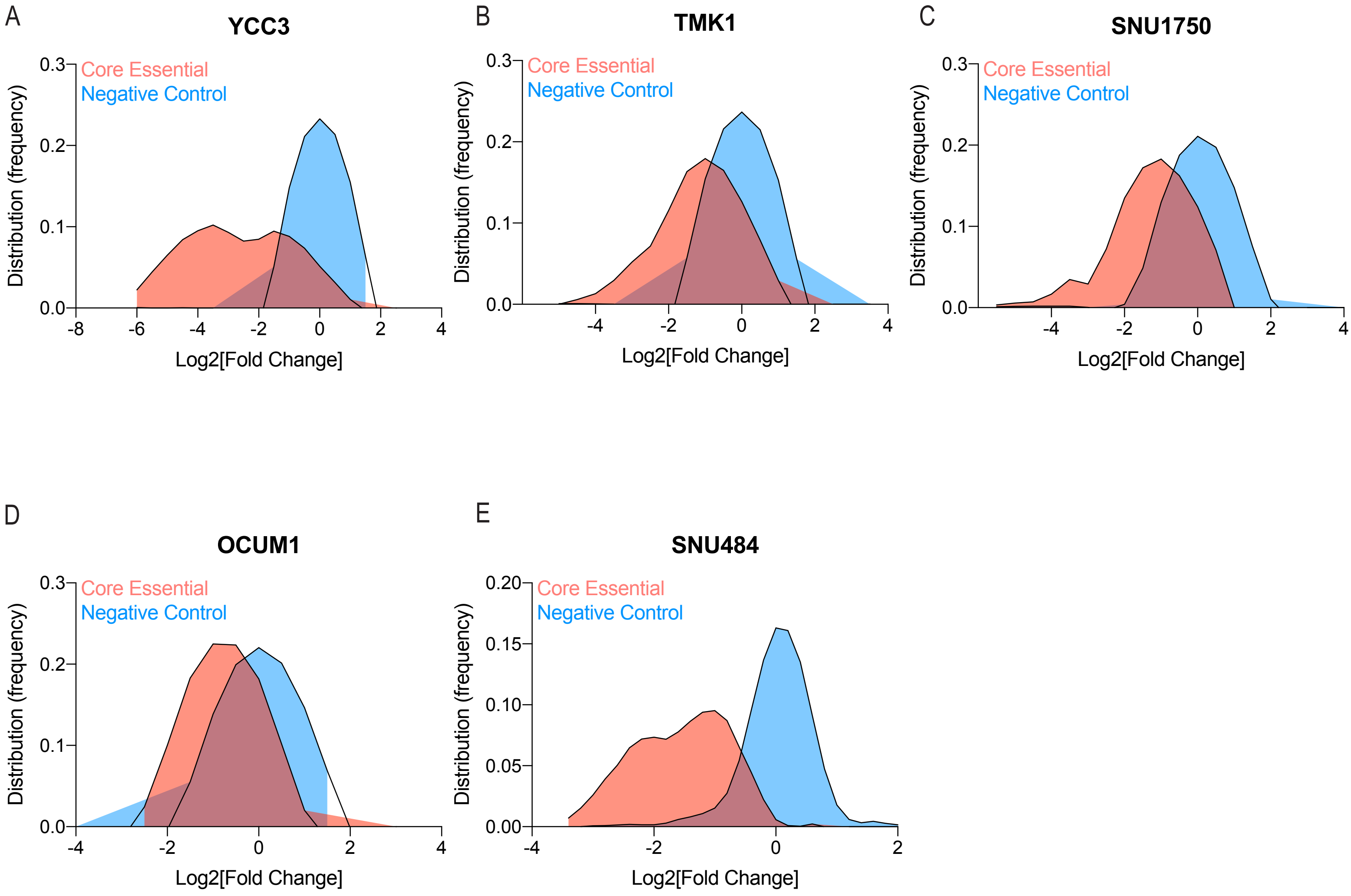
A
YCC3
B
TMK1
C
SNU1750
D
OCUM1
E
SNU484
Core Essential
Negative Control
Distribution (frequency)
Log2[Fold Change]

Figure S2

A

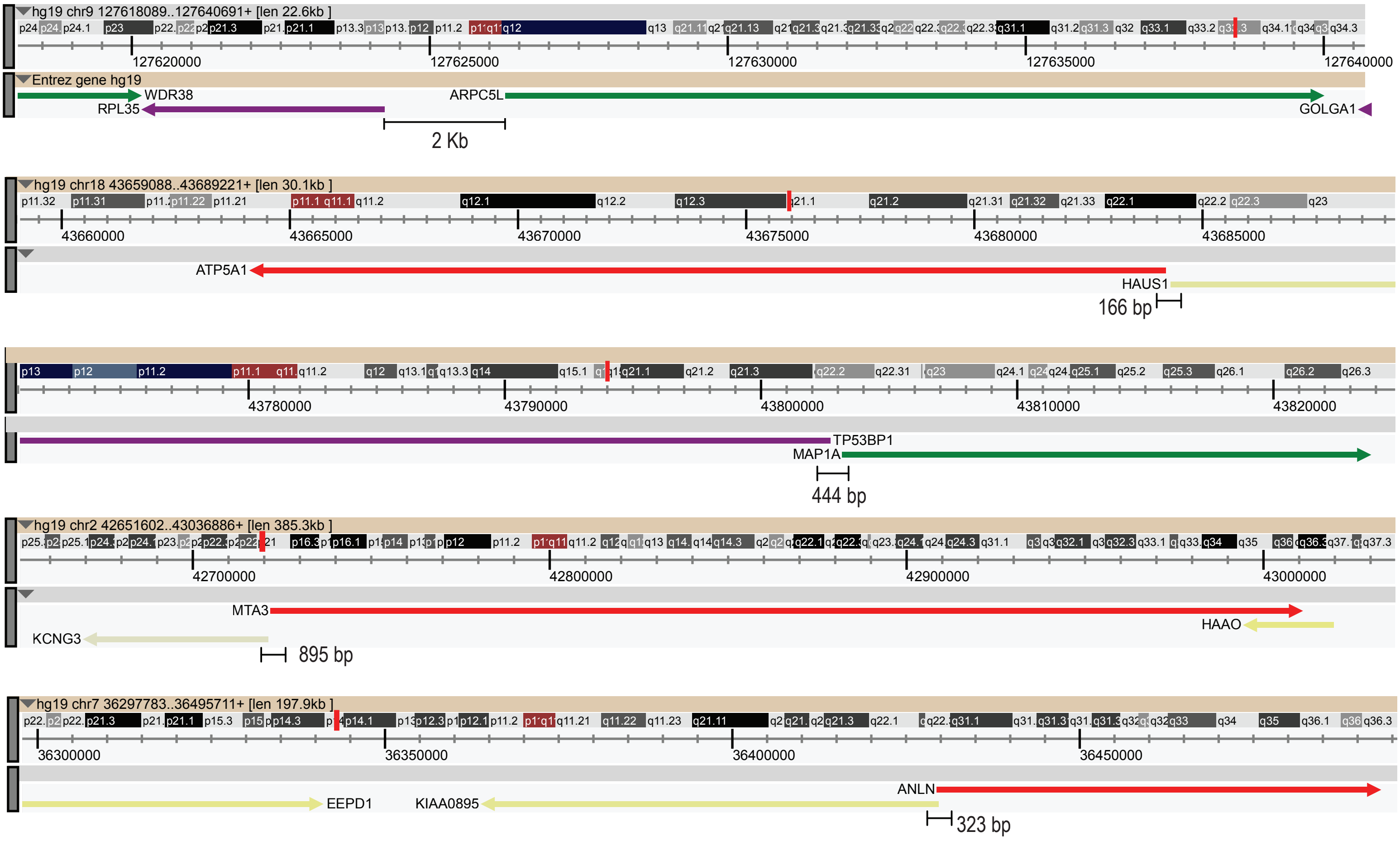
hg19 chr9 127618089..127640691+ [len 22.6kb]
127620000
127625000
127630000
127635000
127640000
Entrez gene hg19
WDR38
RPL35
ARPC5L
GOLGA1
2 Kb
hg19 chr18 43659088..43689221+ [len 30.1kb]
43660000
43665000
43670000
43675000
43680000
43685000
ATP5A1
HAUS1
166 bp
43780000
43790000
43800000
43810000
43820000
TP53BP1
MAP1A
444 bp
hg19 chr2 42651602..43036886+ [len 385.3kb]
42700000
42800000
42900000
43000000
MTA3
HAAO
KCNG3
895 bp
hg19 chr7 36297783..36495711+ [len 197.9kb]
36300000
36350000
36400000
36450000
ANLN
EEPD1
KIAA0895
323 bp

B

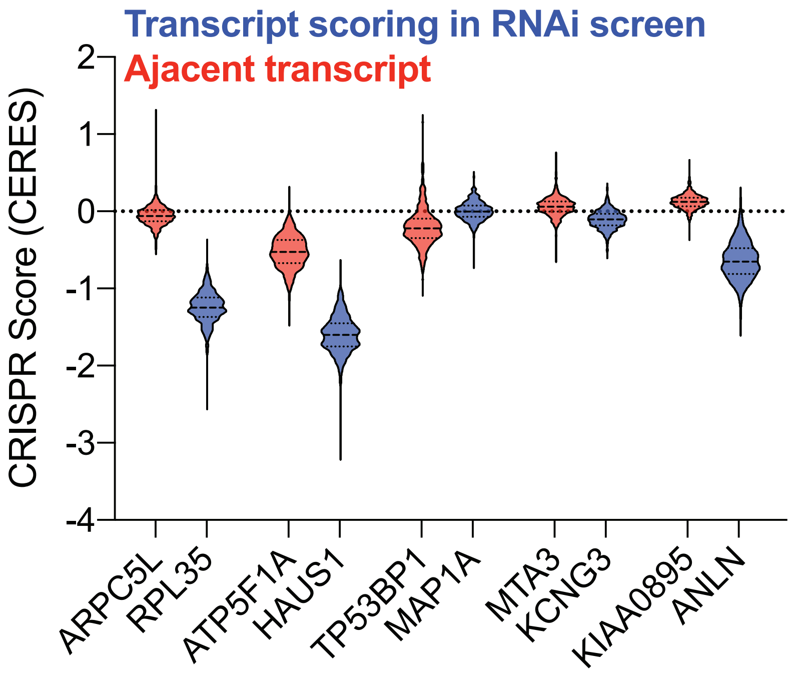
Transcript scoring in RNAi screen
Ajacent transcript
CRISPR Score (CERES)
2
1
0
-1
-2
-3
-4
ARPC5L
RPL35
ATP5F1A
HAUS1
TP53BP1
MAP1A
MTA3
KCNG3
KIAA0895
ANLN

Figure S3

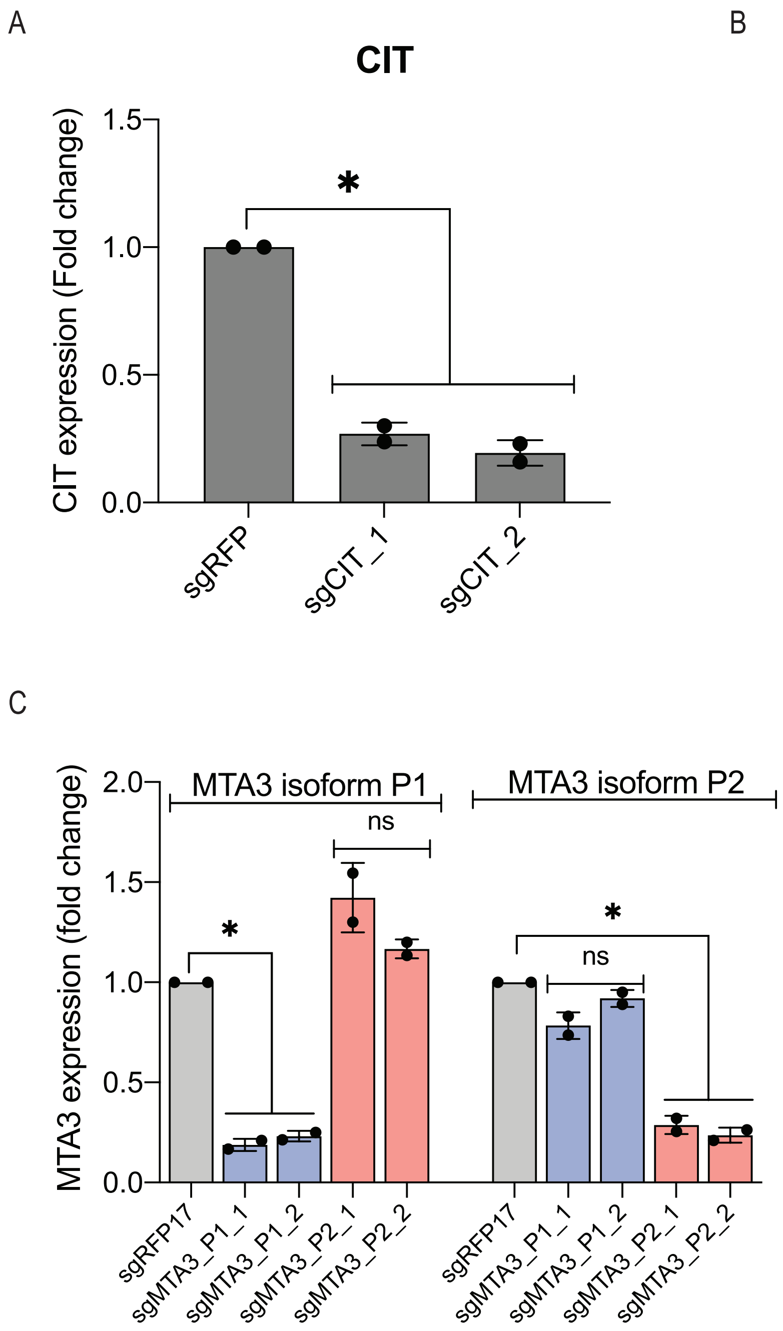
A
CIT
CIT expression (Fold change)
1.5
1.0
0.5
0.0
*
sgRFP
sgCIT_1
sgCIT_2
B
CCNE1
CCNE1 expression (Fold change)
1.5
1.0
0.5
0.0
*
sgRFP
sgCCNE1_1
sgCCNE1_3
C
MTA3 expression (fold change)
2.0
1.5
1.0
0.5
0.0
MTA3 isoform P1
MTA3 isoform P2
ns
*
ns
*
sgRFP17
sgMTA3_P1_1
sgMTA3_P1_2
sgMTA3_P2_1
sgMTA3_P2_2
sgRFP17
sgMTA3_P1_1
sgMTA3_P1_2
sgMTA3_P2_1
sgMTA3_P2_2

Figure S4

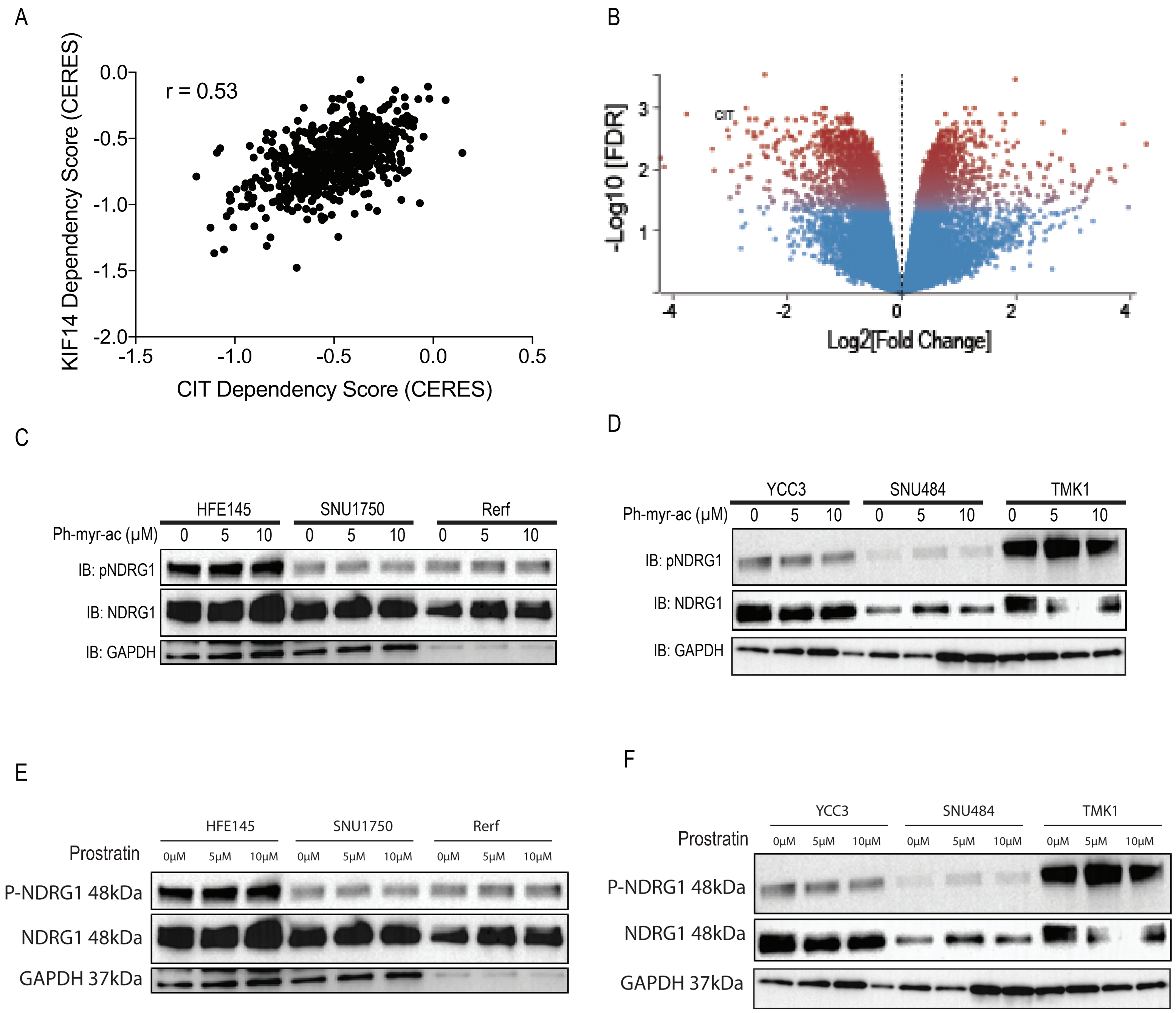
A
r = 0.53
KIF14 Dependency Score (CERES)
CIT Dependency Score (CERES)
B
CIT
-Log10 [FDR]
Log2[Fold Change]
C
HFE145
SNU1750
Rerf
Ph-myr-ac (μM)
IB: pNDRG1
IB: NDRG1
IB: GAPDH
D
YCC3
SNU484
TMK1
Ph-myr-ac (μM)
IB: pNDRG1
IB: NDRG1
IB: GAPDH
E
HFE145
SNU1750
Rerf
Prostratin
0μM 5μM 10μM
P-NDRG1 48kDa
NDRG1 48kDa
GAPDH 37kDa
F
YCC3
SNU484
TMK1
Prostratin
0μM 5μM 10μM
P-NDRG1 48kDa
NDRG1 48kDa
GAPDH 37kDa

Figure S5

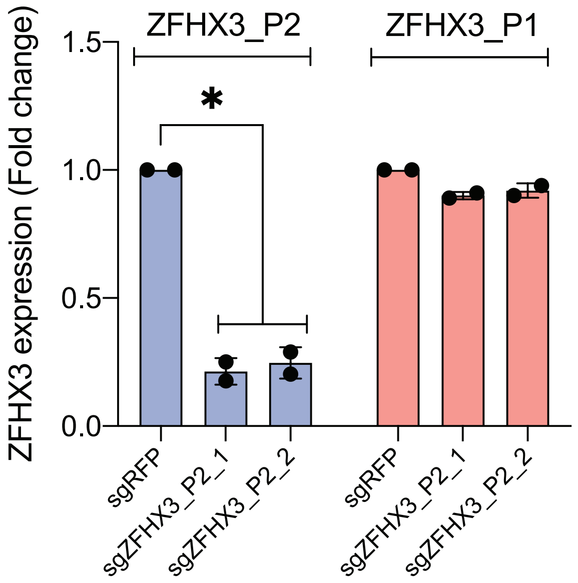
ZFHX3_P2
ZFHX3_P1
*
ZFHX3 expression (Fold change)
1.5
1.0
0.5
0.0
sgRFP
sgZFHX3_P2_1
sgZFHX3_P2_2
sgRFP
sgZFHX3_P2_1
sgZFHX3_P2_2

Figure S6

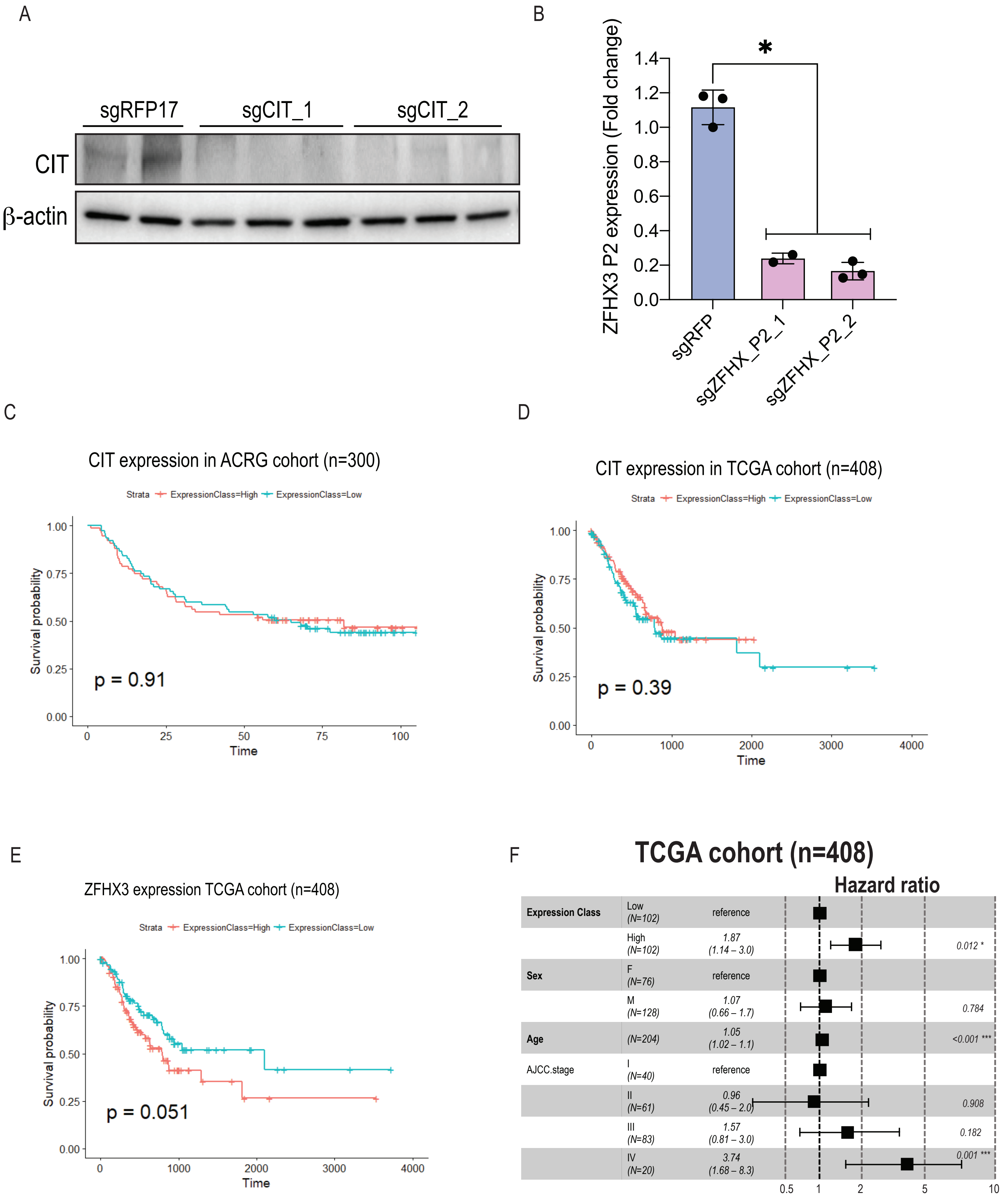
A
sgRFP17
sgCIT_1
sgCIT_2
CIT
β-actin
B
ZFHX3 P2 expression (Fold change)
*
sgRFP
sgZFHX_P2_1
sgZFHX_P2_2
C
CIT expression in ACRG cohort (n=300)
Strata ExpressionClass=High ExpressionClass=Low
Survival probability
p = 0.91
Time
D
CIT expression in TCGA cohort (n=408)
Strata ExpressionClass=High ExpressionClass=Low
Survival probability
p = 0.39
Time
E
ZFHX3 expression TCGA cohort (n=408)
Strata ExpressionClass=High ExpressionClass=Low
Survival probability
p = 0.051
Time
F
TCGA cohort (n=408)
Hazard ratio
Expression Class
Low (N=102) reference
High (N=102) 1.87 (1.14 – 3.0) 0.012 *
Sex
F (N=76) reference
M (N=128) 1.07 (0.66 – 1.7) 0.784
Age (N=204) 1.05 (1.02 – 1.1) <0.001 ***
AJCC.stage
I (N=40) reference
II (N=61) 0.96 (0.45 – 2.0) 0.908
III (N=83) 1.57 (0.81 – 3.0) 0.182
IV (N=20) 3.74 (1.68 – 8.3) 0.001 ***

Figure S7
