# Supplementary material for: CRISPRi enables isoform-specific loss-of-function screens and identification of gastric cancer-specific isoform dependencies
Source: Genome Biol. 2021 Jan 26;22:47. doi: 10.1186/s13059-021-02266-6 (PMC7836456; doi:10.1186/s13059-021-02266-6)
Supplement: Supplementary file 3 — Additional file 3: Review history. [file 13059_2021_2266_MOESM3_ESM.docx]

Review History

**First round of review**

**Reviewer 1**

**Are you able to assess all statistics in the manuscript, including the appropriateness of statistical tests used?** Yes, and I have assessed the statistics in my report.

**Comments to author:**

In this manuscript by Davies et al., the authors perform a CRISPR/Cas9 screen to assess alternative TSS usage in gastric cancer by specifically repressing the TSS of alternative isoforms with dCas9-KRAB. This general research approach and question is exciting, and serves to directly find which alternative transcripts are utilized and lead to altered growth phenotypes in gastric cancer. However, there are multiple severe flaws with this manuscript as it stands that serve to undercut or obscure many of the conclusions. This is due to a lack of any statistics in this manuscript outside of the screen, and the fact that a large set of screen effects are unexplained and could largely be artifacts of library design issues (with highly non-specific guides included) or artifacts from identifying appropriate transcripts for analysis (and essentially just finding differential expression of canonical transcripts instead of true alternative TSSs). Additionally, this manuscript was presented in an extremely confusing fashion due to the lack of detail in both the figures and the legends as well as the methods section. While this review may be long, I detail all of my concerns because many of these can be addressed with the data on-hand and I hope the authors find these comments helpful towards improving their manuscript. These concerns are detailed below:

Major Concerns
1) There is a complete lack of any statistical comparisons in the data from this paper. Multiple comparisons between groups are made for CRISPRi knockdowns (Fig #1B/C/E) and proliferation/CFU assays (Fig #4A/C, Fig #5B, Fig #6F/H) which are central validations of the screen and paper. However no statistics are performed across all of these groups. In fact, there are no statistical comparisons made across the paper at any point besides what was calculated in the initial screen. This is extremely inappropriate and if any conclusions are to be drawn from this data the proper statistical comparisons need to be made. It is further unclear if each replicate is biological or technical. Additionally all bar graphs should be plotted along with individual points for each replicate.

2) The authors propose a bit of structural mechanism behind why ZFHX3 alternative transcripts may have different effects (though this is a generally small exploration and lacks protein-level validation which should be included). However, the mechanistic underpinnings are completely missing with the CIT / ZIC2 / CCNE1 part of the story and I am not entirely convinced, even with some of the extra data on CIT. What is unclear is why does the alternative TSS/isoform repression have a strong growth defect while the other unchanged or canonical isoform does not? Are the alternative isoforms shorter or longer than the predominant canonical transcript? How are the transcripts expressed in comparison with one another (and at what TPM)? I am concerned that the alternative transcript definitions (via H3K4me3) might actually represent the canonical transcript in certain cases. Can you detect differences at the protein level in terms of various the various isoforms? Without this data for all of the genes validated we do not have the full picture of what is happening. A quick Ensembl search showed many inconsistencies with the target transcripts for the screen hits. As far as I can tell the "Gained transcript" for CIT (ENST00000392521) is protein-coding, whereas the  "Unaltered transcript" listed for CIT (ENST00000488203) is not protein-coding and a shorter transcript, which could explain why there seems to be only one band on the Western blot and why there was no effect on growth in the screen for the CIT unaltered transcript (ie. there is no protein of this transcript). When looking up where the unaltered transcript sgRNAs bind, the "unaltered transcript" is near one transcript that could potentially be protein coding (ENST00000612548) and is similar to the P2-labeled PacBio transcript, but it is not the transcript mentioned in the paper, and is apparently not expressed via RNA-seq in any of their cells (from the supplementary tables). Further analysis of ZIC2 and CCNE1 transcripts listed here shows similar trends where the "Gained transcripts" are the protein-coding transcript, while the "Unaltered transcripts" are much shorter and are not protein-coding. As you would expect from the data in Fig#6 which includes protein isoform information, for ZFHX3 both "Gained" and "Unaltered" transcripts are indeed listed as protein-coding. Together, the presentation of screening hit data is very confusing and I have little confidence about the underlying mechanism and that these hits are indeed true positives and represent different regulatory transcripts or different protein isoforms. There are also sgRNA library design issues listed below that further obscure the interpretation of this data.

3) There is a lack of depth in screen validation and issues with library design. The topic of off-targets is not addressed in this manuscript. Importantly it was not mentioned if the authors used a specificity score cutoff while designing the sgRNA library (as explored in Tycko+ Nature Communications 2019). Upon further examination, one of the guides for the ZIC2 "gained transcript" used in this screen seems to have an extremely high degree of off targets with a MM0/MM1/MM2 (MM= mismatch number) values of 79/70/12 according to GTscan! Other ZIC2 gained transcript guides have scores of 0/25/94, 0/0/7, 3/12/4, 43/23/0, and 33/49/3. This is completely unacceptable, as it shows that of these ZIC2 guides in the screen, 66% of guides have multiple 100% sequence mismatches elsewhere in the genome, with some guide targeting as many of 79 locations at once! Similar guide issues occur with some ZFHX3 gained transcripts (1 guide with multiple MM0 mismatches), and ZFHX3 unaltered transcript (which has 3 guides that don't bind anywhere, likely due to 12+mer A-rich sequences).  The authors should go through their screening data and globally throw out any poor quality guides in the analysis that have high degrees of off-targets or other issues. Also it is not clearly noted which guides are used for validation in the manuscript.

While the authors did show isoform specific CRISPRi targeting for a few genes in the first figure of the paper (though without stats), importantly the authors do not show good controls/validations for the core tenet of their screen: that perturbations of alternative TSSs from the screen via CRISPRi do not also effect the expression of the main isoform of the given gene. Isoform-specific expression is not validated for CIT, ZIC2, or CCNE1 and should be added, and in Supplemental Figure #6 the authors examine how each ZFHX3 transcript can be altered by CRISPRi but they do not examine the crosstalk between these perturbations in a similar fashion to how they did in Fig#1 for other genes. This is an important control because CRISPRi targeting to one TSS may be changing expression of the other isoform on the same strand. Also, in the end there are only 4 hits that are validated at any level, and really only two hits that are somewhat thoroughly validated (CIT from Fig #3, ZFHX3 from Fig #6) out of the total 12 hits that were essential in >=2 cell lines. It seems like all of these 12 hits could readily be validated at the functional effect level. Why not follow up on other hits? Also, it seems like the most consistent hit from the screen across all cells was MTA3 - why not follow up on this hit? Without these additional validations it limits the significance of this manuscripts findings. Additionally, why are the FDRs saturated at ~4 for pan-essential genes in HFE145 cells (Fig #2H)?

-Analysis methodology: The methods section for the screen is extremely lacking and I am very concerned about why this data wasn't included. The authors cite their previous paper but that is not sufficient in describing their methods and everything done in this paper should be typed out in the methods. The authors do not clearly specify where was the gRNA library ordered from and how was it cloned, what cell lines were used for the screen, whether the lines were polyclonal or clonal for dCas9-KRAB, what density were the cells seeded and kept at, how many cells were transduced, what plasmid was used for gRNA library expression, if the whole gRNA library was in their plasmid pool, and if it was all represented in their packaged lentivirus (and verified by next gen sequencing of gDNA from early timepoints). The actual sgRNA library pool has 6 guides per transcript but the paper only mentions 5 - which is it? The screen analysis protocol is completely absent in the methods section and the authors do no clearly spell out how thy performed the screen and analysis - instead small details are scattered throughout the manuscript (as I extrapolated above). How many biologic reps were done per screen and cell-type? As far as I can tell it is only one replicate per cell-type, which should be increased at minimum for the healthy gastric cell line. How are data from each replicate combined? What groups are being compared using MAGeCK? What settings are used? Do the authors compare their guides to an early timepoint control transduced population? Importantly the authors need to provide raw counts collected for all of their screening data, as they only provide p-values and logFC values in the supplement.

4) No methods are given for transcript expression experiments that are all over this manuscript. This includes how RNA was extracted, the RNA-seq details (eg. what library prep kit, was it SE or PE, what read length), and a list of the primers used for these experiments (especially for the isoform specific quantification). Moreover it calls into question how the gene expression across this manuscript was even accomplished, which wasn't explained in the text or legends at all. Are the expression measurements in Fig#1 and Supp. Fig #S4 from qPCR or RNA-seq? Was the RNA extracted from the library used for quantification or an individual sgRNA perturbation? There are many issues that I outlined with regards to the lack methods for various parts of the paper specifically, but it is important to mention that the methods section as a whole is extremely lacking depth for nearly every section and is generally confusing with what methods were utilized in different parts of this paper. This makes it difficult to understand exactly what the authors are doing and showing in this work.

Minor Issues:
-Key labels and details are missing from figures and legends. Throughout the figures in the text (and in the figure legends) there are labeling issues which at best greatly limit readability of the manuscript, and at worst completely obscure the results of the study. Generally speaking the figure legends show an extreme lack of detail. Some of these issues are summarized below, but a more thorough reworking of how this data is presented should be required before publication.

---There are many instances in the figures where authors show "fold-change" and then do not clearly state in the figure legends or figures themselves what comparison is being made. This is true for both the screen data and other datasets in the manuscripts.
---Throughout the paper the authors show y-axis labels as "sgRNA Log2FC" when it seems like "Transcript Log2FC" is more appropriate because the authors are averaging all of the guides per transcript (5 sgRNA per transcript
---For Fig#1F (which has a missing label), the figure as presented makes it confusing to show that the authors used a pooled sgRNA library for 55 transcripts and compared their effects on growth as the "fold-change" value. Both of these details should somehow be included in the figure.
---Throughout the paper multiple cell lines are shown interchangeably but there is no consistent usage of one cell line throughout. As such, it begins to be very hard to track what is going on and why by just reading the figures/legends. Further it's hard to keep track of which cell lines/data are normal versus GC related (when the authors introduce HFE145 normal gastric cell line). In the figures the transformed versus not transformed cell lines should be made clear
---The description of the screen in the figure and legends is lacking. Important details such as the type of screen (growth depletion), cell-types used, # of guides/Transcript, and analysis comparisons should be included.
---In Fig#2B, the H3K4me3 data is zoomed on alternative TSS, which the authors use to show the utilization of an alternative transcript/TSS in gastric cancers. However, the tracks do not show that the H3K4me3 looks like at the typical/normal/healthy TSS. Further, the "gain" in H3K4me3 at this transcript doesn't seem to be any higher than the H3K4me3 signal ~2kb downstream that is not associated with any TRPM2 transcript. I suspect that this degree of signal is largely background compared to the typical TSS levels, and an inset should be shown of the H3K4me3 at the usual TSS. This further feeds into other questions raised above about the true functional effects of most of these alternative TSS's and the absolute magnitude of change of expression.

-For experiments where CRISPRi experiments are performed, the figure labels need to be more clear that this is a CRISPRi experiment rather than just listing "sgRNAXX_P1_1", as this data on quick glance can be interpreted as a CRISPRko experiment or any other Cas9-related perturbation. Further labeling the validations as "sgCIT" is misleading, as the guide targets the gained isoform rather than the normal isoform.

-Where are the loci for Fig#3F-H/Fig#6B and what are the "P200085" labels? No explanation of what exactly these labels represent are made, such that  interpreting this data is difficult. Perhaps the authors should show a map of where these regions are on the genome track.  

-Fig#7 and Fig#4 are too small and incidental to be their own figures. Fig #7 should be part of Fig#6 and Fig #4 should likely be in Figure #3 as they are directly related. Fig #3F-H should likely be in the supplement due to the lack of rigor in these comparisons (no stats, error bars, etc.)

-Typos throughout figures and paper.

**Reviewer 2**

**Are you able to assess all statistics in the manuscript, including the appropriateness of statistical tests used?** No, I do not feel adequately qualified to assess the statistics.

**Comments to author:**

First of all, it is not clear in the organization of this article. The authors stated that CIT kinase as a novel GC dependency, however, they focused on the genes which express isoforms with opposite functions and used ZFHX3 as an example.
Secondly, I think the statistical result shown in Figure 4C can't accurately reflect the situation in Figure 4B. The figures and statistical results are not corresponding.
Further, you only focus on genes that affect the cloning of GC cells, not the proliferation of GC cells. This paper needs to supplement experiments that reflect cell proliferation, such as EdU or BRDU. In addition,  I think the authors should confirm this results in vivo.
The paper lack the further molecular mechanism explore of ZFHX3 about its two isoforms' functional role and clinical importance. The authors should add the experiments to  explore how the two isoforms of ZFHX3 play oppose functions in GC patients.

**Authors Response**

Point by point response to reviewers

We would like to thank the reviewers and specially reviewer 1 for the insightful, positive and constructive comments. In the revised manuscript we have addressed all the concerns raised by the reviewers and have added additional experiments including in vivo studies (Fig. 7), BrDU proliferation assays (Fig. 4 and 6) and follow-up mechanistic studies looking at ZFHX3 downstream target genes (Fig. 6). We have also addressed the reviewers concern about sgRNA specificity and have reanalysed our screening data after filtering out all sgRNAs that had more than one perfect alignment to the human genome. This has not changed the main conclusions of our manuscript however, this resulted in one of our previous hits (ZIC2) to not pass our threshold as a hit. We hope that we have addressed all the reviewers concerns and that you find the revised manuscript suitable for publication.

**Reviewer 1:**

Reviewer #1: In this manuscript by Davies et al., the authors perform a CRISPR/Cas9 screen to assess alternative TSS usage in gastric cancer by specifically repressing the TSS of alternative isoforms with dCas9-KRAB. This general research approach and question is exciting, and serves to directly find which alternative transcripts are utilized and lead to altered growth phenotypes in gastric cancer. However, there are multiple severe flaws with this manuscript as it stands that serve to undercut or obscure many of the conclusions. This is due to a lack of any statistics in this manuscript outside of the screen, and the fact that a large set of screen effects are unexplained and could largely be artifacts of library design issues (with highly non-specific guides included) or artifacts from identifying appropriate transcripts for analysis (and essentially just finding differential expression of canonical transcripts instead of true alternative TSSs). Additionally, this manuscript was presented in an extremely confusing fashion due to the lack of detail in both the figures and the legends as well as the methods section. While this review may be long, I detail all of my concerns because many of these can be addressed with the data on-hand and I hope the authors find these comments helpful towards improving their manuscript. These concerns are detailed below:

We thank the reviewer for the constructive comments and have modified the manuscript extensively to answer all of the reviewers’ concerns. In addition to adding more information of the sgRNA selection and more validation experiments (see details below) we modified the figure legend so it is clearer. We hope that the reviewer finds the revised manuscript more suitable.

Major Concerns

1) There is a complete lack of any statistical comparisons in the data from this paper. Multiple comparisons between groups are made for CRISPRi knockdowns (Fig #1B/C/E) and proliferation/CFU assays (Fig #4A/C, Fig #5B, Fig #6F/H) which are central validations of the screen and paper. However no statistics are performed across all of these groups. In fact, there are no statistical comparisons made across the paper at any point besides what was calculated in the initial screen. This is extremely inappropriate and if any conclusions are to be drawn from this data the proper statistical comparisons need to be made. It is further unclear if each replicate is biological or technical. Additionally all bar graphs should be plotted along with individual points for each replicate.

We thank the reviewer for this comment and have added to all the relevant figures statistical analysis as well as bar graphs with all replicate points. In addition, we have updated the figure legends which now have a detailed explanation of how the data set was generated.

2) The authors propose a bit of structural mechanism behind why ZFHX3 alternative transcripts may have different effects (though this is a generally small exploration and lacks protein-level validation which should be included).

We have extensively tried to find an Ab that could detect endogenous ZFHX3 protein (see Fig. 1 below for an example). Although we could detect robust expression of ZFHX3 using qPCR we could not find an Ab that is able to detect endogenous ZFHX3 protein. The best Ab we were able to find (Abcam #119909) could detect exogenously over-expressed ZFHX3 but not endogenous ZFHX3.

To address the reviewers concerns and to get a better understanding and validation of ZFHX3 we included several new experiments that further validate and give some mechanistic insights into mechanism of action these include:

a) In-vivo validation in xenograft models of ZFHX3 isoform P2 as essential for growth of gastric cancers (revised Fig. 7A,B).
b) BrDU proliferation assay demonstrating that suppression of ZFHX3 isoform P2 results in proliferation arrest (revised Fig. 6G).
c) Quantification of known ZFHX3 target genes (*CDKN1A* and *MYB*) following CRISPRi mediated suppression of transcript P2 shows the opposite effect on the expression of previously published ZFHX3 target genes.

Combined all of this data suggest a mechanism of action for ZFHX3 isoform P2 that is different then isoform P1. Specifically, our data shows that the P2 isoform of ZFHX3 functions as an oncogene rather than a tumour suppressor and suggest a feedback loop mechanism. Importantly this could only be identified using a promoter specific CRISPRi library and would not be found using commonly used CRISPR libraries.

Figure 1: ZFHX3 Ab staining. Western blot analysis of HEK293FT cells transfected with a ZFHX3 over expression construct (Addgene #40927) or protein lysates from YCC3 cells stained with a ZFHX3 Ab (Abcam #119909) demonstrating that commercially available Ab could not detect endogenous ZFHX3 protein.

However, the mechanistic underpinnings are completely missing with the CIT / ZIC2 / CCNE1 part of the story and I am not entirely convinced, even with some of the extra data on CIT. **What is unclear is why does the alternative TSS/isoform repression have a strong growth defect while the other unchanged or canonical isoform does not**? Are the alternative isoforms shorter or longer than the predominant canonical transcript? How are the transcripts expressed in comparison with one another (and at what TPM)? I am concerned that the alternative transcript definitions (via H3K4me3) might actually represent the canonical transcript in certain cases. **Can you detect differences at the protein level in terms of various the various isoforms?** Without this data for all of the genes validated we do not have the full picture of what is happening. A quick Ensembl search showed many inconsistencies with the target transcripts for the screen hits. As far as I can tell the "Gained transcript" for CIT (ENST00000392521) is protein-coding, whereas the "Unaltered transcript" listed for CIT (ENST00000488203) is not protein-coding and a shorter transcript, which could explain why there seems to be only one band on the Western blot and why there was no effect on growth in the screen for the CIT unaltered transcript (ie. there is no protein of this transcript).

When looking up where the unaltered transcript sgRNAs bind, the "unaltered transcript" is near one transcript that could potentially be protein coding (ENST00000612548) and is similar to the P2-labeled PacBio transcript, but it is not the transcript mentioned in the paper, and is apparently not expressed via RNA-seq in any of their cells (from the supplementary tables). Further analysis of ZIC2 and CCNE1 transcripts listed here shows similar trends where the "Gained transcripts" are the protein-coding transcript, while the "Unaltered transcripts" are much shorter and are not protein-coding.

We are sorry for the confusion in presenting our results. In most cases we found that the essential alternative transcript is the major expressed transcript. However, in some, more rare cases like *ZFHX3* and *HNF4A* both transcripts are expressed. In this paper we defined canonical transcripts based on NCBI annotations. The reviewer has a valid point and we have definitely observed that many times NCBI annotations do not point at the expressed transcript even for very well-known genes such as *MYC*. To clarify this point, we have modified the name of the alternative transcripts to gastric cancer gained transcripts and unaltered transcripts. We have also modified the text to clarify that in most of these cases the gastric cancer gained transcript is the major expressed transcript.

3) There is a lack of depth in screen validation and issues with library design. The topic of off-targets is not addressed in this manuscript. Importantly it was not mentioned if the authors used a specificity score cutoff while designing the sgRNA library (as explored in Tycko+ Nature Communications 2019). Upon further examination, one of the guides for the ZIC2 "gained transcript" used in this screen seems to have an extremely high degree of off targets with a MM0/MM1/MM2 (MM= mismatch number) values of 79/70/12 according to GTscan! Other ZIC2 gained transcript guides have scores of 0/25/94, 0/0/7, 3/12/4, 43/23/0, and 33/49/3. This is completely unacceptable, as it shows that of these ZIC2 guides in the screen, 66% of guides have multiple 100% sequence mismatches elsewhere in the genome, with some guide targeting as many of 79 locations at once! Similar guide issues occur with some ZFHX3 gained transcripts (1 guide with multiple MM0 mismatches), and ZFHX3 unaltered transcript (which has 3 guides that don't bind anywhere, likely due to 12+mer A-rich sequences). The authors should go through their screening data and globally throw out any poor quality guides in the analysis that have high degrees of off-targets or other issues. Also it is not clearly noted which guides are used for validation in the manuscript.

In our original manuscript we have included many sgRNAs that had multiple perfect matches. This was done since for many of these promoters we only found a limited number of PAM sites. Based on the reviewers’ suggestion we have reanalysed our data after exclusion of any sgRNA that had multiple exact match’s (total of 1,700 sgRNAs excluded). This filtering removed ZIC2 from our hits but most of our top scoring hits still scored (see revised Fig. 3A and B). Accordingly, we removed ZIC2 from the manuscript. Due to this filtering the sgRNAs targeting isoform P1 of ZFHX3 no longer score as TSG. Although we have validated these sgRNAs and showed that they are able to supress the expression of this isoform and induce a TSG phenotype we have decided to remove the experiments that show this due to the possible off target effects of these sgRNAs. In addition, we have updated the methods section and added the sequences of all the sgRNAs used in validation (Supplementary table S7).

While the authors did show isoform specific CRISPRi targeting for a few genes in the first figure of the paper (though without stats), importantly the authors do not show good controls/validations for the core tenet of their screen: that perturbations of alternative TSSs from the screen via CRISPRi do not also effect the expression of the main isoform of the given gene. Isoform-specific expression is not validated for CIT, ZIC2, or CCNE1 and should be added, and in Supplemental Figure #6 the authors examine how each ZFHX3 transcript can be altered by CRISPRi but they do not examine the crosstalk between these perturbations in a similar fashion to how they did in Fig#1 for other genes. This is an important control because CRISPRi targeting to one TSS may be changing expression of the other isoform on the same strand.

We thank the reviewer for this comment. As stated above for many of these transcripts we found very low expression of the non-essential transcript which we are not able to detect using traditional approaches.

Also, in the end there are only 4 hits that are validated at any level, and really only two hits that are somewhat thoroughly validated (CIT from Fig #3, ZFHX3
from Fig #6) out of the total 12 hits that were essential in >=2 cell lines. It seems like all of these 12 hits could readily be validated at the functional effect level. Why not follow up on other hits? Also, it seems like the most consistent hit from the screen across all cells was MTA3 - why not follow up on this hit? Without these additional validations it limits the significance of this manuscripts findings. Additionally, why are the FDRs saturated at ~4 for pan-essential genes in HFE145 cells (Fig #2H)?

We chose to validate those three genes based on the fact that they were essential in the GC cell lines and not essential in normal gastric cell line (HFE145). As suggested by the reviewer we also added validation of MTA3 which had a strong phenotype in 2D cultures (Revised Fig. 4). The saturation of the FDR is due to the library size. The FDR is calculated by correcting for multiple hypothesis and is those capped by the number of hypothesis that are tested.

-Analysis methodology: The methods section for the screen is extremely lacking and I am very concerned about why this data wasn't included. The authors cite their previous paper but that is not sufficient in describing their methods and everything done in this paper should be typed out in the methods. The authors do not clearly specify where was the gRNA library ordered from and how was it cloned, what cell lines were used for the screen, whether the lines were polyclonal or clonal for dCas9-KRAB, what density were the cells seeded and kept at, how many cells were transduced, what plasmid was used for gRNA library expression, if the whole gRNA library was in their plasmid pool, and if it was all represented in their packaged lentivirus (and verified by next gen sequencing of gDNA from early timepoints). The actual sgRNA library pool has 6 guides per transcript but the paper only mentions 5 - which is it? The screen analysis protocol is completely absent in the methods
section and the authors do no clearly spell out how thy performed the screen and analysis - instead small details are scattered throughout the manuscript (as I extrapolated above). How many biologic reps were done per screen and cell-type? As far as I can tell it is only one replicate per cell-type, which should be increased at minimum for the healthy gastric cell line. How are data from each replicate combined? What groups are being compared using MAGeCK? What settings are used? Do the authors compare their guides to an early timepoint control transduced population? Importantly the authors need to provide raw counts collected for all of their screening data, as they only provide p-values and logFC values in the supplement.

We have extensively modified the methods section which now includes details of replicates and MAGeCK analysis.

4) No methods are given for transcript expression experiments that are all over this manuscript. This includes how RNA was extracted, the RNA-seq details (eg. what library prep kit, was it SE or PE, what read length), and a list of the primers used for these experiments (especially for the isoform specific quantification). Moreover it calls into question how the gene expression across this manuscript was even accomplished, which wasn't explained in the text or legends at all. Are the expression measurements in Fig#1 and Supp. Fig #S4 from qPCR or RNA-seq? Was the RNA extracted from the library used for quantification or an individual sgRNA perturbation? There are many issues that I outlined with regards to the lack methods for various parts of the paper specifically, but it is important to mention that the methods section as a whole is extremely lacking depth for nearly every section and is generally confusing with what methods were utilized in different parts of this paper.
This makes it difficult to understand exactly what the authors are doing and showing in this work.

We are sorry for any confusion and have added these sections to the methods as well as to the figure legends.

Minor Issues:

-Key labels and details are missing from figures and legends. Throughout the figures in the text (and in the figure legends) there are labeling issues which at best greatly limit readability of the manuscript, and at worst completely obscure the results of the study. Generally speaking the figure legends show an extreme lack of detail. Some of these issues are summarized below, but a more thorough reworking of how this data is presented should be required before publication.

We are sorry for this and have extensively corrected the figure legend which is now very detailed.

---There are many instances in the figures where authors show "fold-change" and then do not clearly state in the figure legends or figures themselves what comparison is being made. This is true for both the screen data and other datasets in the manuscripts.

We have modified the figure legends which now explain this in detail.

---Throughout the paper the authors show y-axis labels as "sgRNA Log2FC" when it seems like "Transcript Log2FC" is more appropriate because the authors are averaging all of the guides per transcript (5 sgRNA per transcript

These figures show all the individual sgRNAs that target a particular transcript and are not an average.

---For Fig#1F (which has a missing label), the figure as presented makes it confusing to show that the authors used a pooled sgRNA library for 55 transcripts and compared their effects on growth as the "fold-change" value. Both of these details should somehow be included in the figure.

We have corrected these mistakes and have updated the figure legend.

---Throughout the paper multiple cell lines are shown interchangeably but there is no consistent usage of one cell line throughout. As such, it begins to be very hard to track what is going on and why by just reading the figures/legends. Further it's hard to keep track of which cell lines/data are normal versus GC related (when the authors introduce HFE145 normal gastric cell line). In the figures the transformed versus not transformed cell lines should be made clear

We are sorry for the confusion and have added more detail in the figure legend and throughout the text to clarify that HFE145 is a normal immortalised gastric cell line

---The description of the screen in the figure and legends is lacking. Important details such as the type of screen (growth depletion), cell-types used, # of guides/Transcript, and analysis comparisons should be included.

As requested by the reviewer we have added these details to the legend.

---In Fig#2B, the H3K4me3 data is zoomed on alternative TSS, which the authors use to show the utilization of an alternative transcript/TSS in gastric cancers. However, the tracks do not show that the H3K4me3 looks like at the typical/normal/healthy TSS. Further, the "gain" in H3K4me3 at this transcript doesn't seem to be any higher than the H3K4me3 signal ~2kb downstream that is not associated with any TRPM2 transcript. I suspect that this degree of signal is largely background compared to the typical TSS levels, and an inset should be shown of the H3K4me3 at the usual TSS. This further feeds into other questions raised above about the true functional effects of most of these alternative TSS's and the absolute magnitude of change of expression.

The TSS signals shown in Fig. 2B are an average signal from 17 tumour and normal pairs and have been published in full (Qamara et al. Cancer Discovery 2017). These experiments use nano-chip seq to identify H3K4me3 signals and have been extensively validated (see above paper). Furthermore, we demonstrate in this manuscript that these signals predict the expression of active gastric cancer related transcripts.

-For experiments where CRISPRi experiments are performed, the figure labels need to be more clear that this is a CRISPRi experiment rather than just listing "sgRNAXX_P1_1", as this data on quick glance can be interpreted as a CRISPRko experiment or any other Cas9-related perturbation. Further labeling the validations as "sgCIT" is misleading, as the guide targets the gained isoform rather than the normal isoform.

We thank the reviewer for this comment and have modified the figure accordingly.

-Where are the loci for Fig#3F-H/Fig#6B and what are the "P200085" labels? No explanation of what exactly these labels represent are made, such that interpreting this data is difficult. Perhaps the authors should show a map of where these regions are on the genome track.

We have modified the figure and explained in more detail the legends.

-Fig#7 and Fig#4 are too small and incidental to be their own figures. Fig #7 should be part of Fig#6 and Fig #4 should likely be in Figure #3 as they are directly related. Fig #3F-H should likely be in the supplement due to the lack of rigor in these comparisons (no stats, error bars, etc.)

We have modified the figures and in the revised manuscript now Fig 7 describes in vivo experiments.

-Typos throughout figures and paper.

We are sorry for these typos that have been corrected in the revised manuscript.

**Reviewer #2:**

First of all, it is not clear in the organization of this article. The authors stated that CIT kinase as a novel GC dependency, however, they focused on the genes which express isoforms with opposite functions and used ZFHX3 as an example.

The paper focuses on validation of two examples CIT and ZFHX3 which represent two types of dep. Figure 5 focuses on CIT showing mechanism and potential small molecule inhibitors. Figure 6 of the paper describes ZFHX3. In addition, we have added new data including xenograft studies and BrDU proliferation assays.

Secondly, I think the statistical result shown in Figure 4C can't accurately reflect the situation in Figure 4B. The figures and statistical results are not corresponding. Further, you only focus on genes that affect the cloning of GC cells, not the proliferation of GC cells. This paper needs to supplement experiments that reflect cell proliferation, such as EdU or BRDU.

We are sorry for the confusion. Fig 4C and 4B are different experiments. Fig. 4B is a crystal violet staining assay which is a 2D proliferation assay. Fig. 4C (which is Fig. 4D in the revised manuscript) is an anchorage independent growth assay (3D). We have revised the figure legend to explain these in more detail. As requested by the reviewer we have added BrDU assays as another approach to validate the proliferation arrest phenotype induced by suppression of these transcripts (Fig. 4C and 6G in the revised manuscript).

In addition, I think the authors should confirm this results in vivo.

As requested by the reviewer we performed in vivo xenograft experiments for validation of CIT, ZFHX3. These results further validate our initial results and are now added to the new figure 7 of the revised manuscript.

The paper lack the further molecular mechanism explore of ZFHX3 about its two isoforms' functional role and clinical importance. The authors should add the experiments to explore how the two isoforms of ZFHX3 play oppose functions in GC patients.

To gain insights into the mechanism of the oncogenic form of ZFHX3 we used qPCR looking at two known ZFHX3 target genes *CDNKN1A* which is up regulated by the TSG form of ZFHX3 and *MYB* which is downregulated by the TSG form of ZFHX3. We found that CRISPRi mediated suppression of transcript P2 shows the opposite effect on the expression of previously published ZFHX3 target genes. All of our data suggest a mechanism of action for ZFHX3 isoform P2 that is different than isoform P1. Specifically, our data shows that the P2 isoform of ZFHX3 functions as an oncogene rather than a tumour suppressor and suggest a feedback loop mechanism. Importantly this could only be identified using a promoter specific CRISPRi library and would not be found using commonly used CRISPR libraries.

**Second round of review**

**Reviewer 1**

The authors have addressed many of my concerns in this revision, and I appreciate the considerable work done in this revision. I found the in vivo data added in this revision to greatly strengthen the paper (and it seems like quite a bit of work for a revision!). However, I feel like there are still a few remaining issues with how the data is currently presented that were not fully addressed in this revision. This presentation hinders the impact and interpretation of the manuscript and as such should be further clarified prior to publication (and besides issue #2, shouldn’t require any new data or drastic changes).

Issue #1: The central principle behind this paper is that there are isoform-specific transcription differences in gastric cancer that drive progression of the diseased state, and further that CRISPRi library screening can be used to query alternate isoform- expression. However, as shown, there is not enough information to understand and anchor the isoform-specific differences in a meaningful way (which I had mentioned in my first review and remained unaddressed). For each isoform studied it would be useful to show in a table format: a) what are the exact ENST IDs for each isoform, b) what are the differences in TPM between the isoforms being compared in the studies, c) if the transcript is protein coding and how long the protein is, and d) does the gained transcript represent the canonical TSS or an alternative TSS. As detailed more below, I am greatly confused about when the authors are showing a gastric cancer-specific increase in the canonical gene TSS and when the authors show gastric cancer-specific transcription from an alternative promoter/TSS. Further the authors frequently refer to transcripts that are never expressed in these gastric cancer lines. When filling these criteria out in my head I found a few issues:

A)     Isoform-specific expression is frequently compared with non-expressed/non-protein coding transcripts which is not a relevant comparison. CIT and CCNE1 “unaltered transcripts” are not protein-coding and it is unclear at what TPM they are expressed in these cells (the authors mention that in some cases they aren’t expressed at all). The authors mention that this is a control for the specificity of this approach, but their inclusion as a comparison thus does not make much sense to me and generally seems like a strawman comparison (eg. comparing any canonical TSS/transcript to a non-expressed/non-protein-coding transcript would technically show isoform-specific differences but not have any functional consequence).

B)     Three out of the four isoform-specific comparisons made in the paper seem to be just a GC-specific increase in the canonical TSS, which is obscured in the presentation of the data. With respect to canonical TSS identification, I agree with the authors that it can be difficult to ascertain based on various sources. However, another way of framing their isoform-specific CRISPRi screening approach is to answer the same question the authors pose in their manuscript: if someone used a common genome-wide CRISPRi library (ie. the CRISPRi Dolcetto library from the Doench Lab) would they have queried the ‘gained isoform’ the authors identified in their screen? For CIT/CCNE1/MTA1 the answer is yes, as all of the guides in this manuscript fall in the same region as the guides from the Dolcetto library. This indicates that the “isoform-specific differences” that the author mention for these genes are largely just the canonical transcript being turned on in the disease state (and there is no evidence that other transcripts are expressed at relevant levels). However, for ZFHX3 the answer is that Dolcetto only examines the P1 transcript guides and not the P2 transcript guides, highlighting a very interesting and important part of this approach; that the authors can examine non-canonical transcript usage in one screen.

As such the manuscript should be modified to reflect that the hits in CIT/CCNE1/MTA1 are likely from “canonical TSS” activation/transcription (and don’t really reflect alternative isoform usage), and that the results from ZFHX3 are from non-canonical alternative TSSs that become activated in gastric cancer. I would argue that only the ZFHX3 case truly reflects the title and abstract of this paper, while the CIT/CCNE1/MTA1 cases are just cases involving the canonical isoform being turned on without any significant influences from the alternative isoforms. Importantly, this does not mute the impact of the CIT/CCNE1/MTA1 work in the paper as they are still interesting and relevant hits, however the isoform-specific usage story for these transcripts needs to be clarified and corrected in revision of the text/abstract.
Issue #2: The authors provide no extra validation of the core tenet of their approach that I mentioned in my first review, which was that perturbations of alternative TSSs for different isoforms from the screen via CRISPRi does not also effect the expression of the main isoform of the given gene. While they performed this experiment in Figure #1 for HNF4A, these dCas9 perturbations are loci-specific and need to be repeated for other transcripts found in the screen. In response to this original query, the authors mentioned that they were unable to detect certain transcripts, but this point only confuses me further. The authors quantified ZFHX3 P1 expression in the first version of this manuscript (which is now removed in this revision) in addition to ZFHX3 P2 isoform expression. RNA-seq data from both TCGA and this manuscript (in the supplement) suggests that both ZFHX3 transcripts should be relatively evenly expressed and present in detectable amounts. So I am still unclear why the authors can’t use a healthy or diseased gastric line with ZFHX3 P1 expression for this validation. I state this point again because I am not sure whether the P2 transcript can be perturbed without effecting the P1 transcript (due to P2 being the shorter transcript), as many studies have shown that intronic dCas9 binding can reduce transcript levels in a strand-specific fashion by displacing RNAP2 (Qi+ Cell 2013, for example).

As a side-note, the authors might want to highlight in the discussion that the extended N-terminus of the ZFHX3 protein P2 isoform may represent an opportunity for different mutational control during cancer that could potentially play a role in. the shift of TSG functionality to oncogenic functionality.
Issue #3: The authors run into the same issue with sgRNA specificity with the newly-included MTA3 results, where sgMTA3_P2_2 has multiple off-targets including one with a 100% match on chr17. This worries me as the authors mentioned in their response to reviewers that they removed any guides that had multiple exact matches in this revision, and as such this guide shouldn’t even be included in the analysis.

Issue #4: I am unsure if it is completely appropriate to perform statistical comparisons on samples that have n=2 (and technically n=1 for each guide used in Figure#4H), though I will leave this point up to the editor’s discretion and statistical guidelines of the journal.  

Small points to correct/add:
-There are currently no text labels/legends for the supplemental tables
-Supplemental Table 5/6 are missing.
-All bar graphs should ideally show data points, as some of the figures have shown these while others have not.
-Figure #2H-M is not colorblind friendly
-Figure #3C-E tpyo for “unaltered”
-Mentions of the “ZFHX3 promoter” in the text without references to which isoform.

**Authors Response**

**Point-by-point responses to the reviewers’ comments:**

Reviewer #1: The authors have addressed many of my concerns in this revision, and I appreciate the considerable work done in this revision. I found the in vivo data added in this revision to greatly strengthen the paper (and it seems like quite a bit of work for a revision!). However, I feel like there are still a few remaining issues with how the data is currently presented that were not fully addressed in this revision. This presentation hinders the impact and interpretation of the manuscript and as such should be further clarified prior to publication (and besides issue #2, shouldn’t require any new data or

drastic changes).

Issue #1: The central principle behind this paper is that there are isoform-specific transcription differences in gastric cancer that drive progression of the diseased state, and further that CRISPRi library screening can be used to query alternate isoform-expression. However, as shown, there is not enough information to understand and anchor the isoform-specific differences in a meaningful way (which I had mentioned in

my first review and remained unaddressed). For each isoform studied it would be useful to show in a table format: a) what are the exact ENST IDs for each isoform, b) what are the differences in TPM between the isoforms being compared in the studies, c) if the transcript is protein coding and how long the protein is, and d) does the gained transcript represent the canonical TSS or an alternative TSS. As detailed more below, I am greatly confused about when the authors are showing a gastric cancer-specific increase in the canonical gene TSS and when the authors show gastric cancer-specific transcription from an alternative promoter/TSS. Further the authors frequently refer to transcripts that are never expressed in these

gastric cancer lines. When filling these criteria out in my head I found a few issues:

- We thank the reviewer about this comment and have modified Table S3 to make this clearer. The revised Table S3 has the, ESTN ID, TPM values (average) and information about protein coding (protein ID and transcript type). We agree with the reviewer that the definition of what is a canonical or non-canonical transcript is confusing. In fact, it is really difficult to pinpoint or define what is a canonical transcript. As we have shown, although the most expressed transcript is a good indicator for its essentiality (see our previous paper PMID: 28534478) it is definitely not always the case. For example, we show in Fig. 1 that the levels of the P1 transcript from HNF4A are higher than P2 however, the P2 transcript transcribes the active HNF4A protein. In our new Table S3 you could find many more examples. For this reason, we decided to move from these definitions and use the terms altered (transcripts that are gained/lost in cancer) or unaltered (transcripts that do not show any difference between cancer and normal tissue). To address the reviewers comments regarding transcripts that are

not expressed we have modified the text which now explicitly says that CIT and CCNE1 are gained transcripts in which only one isoform is expressed and MTA3 and ZFHX3 represent genes in which an additional transcript is gained.

A) Isoform-specific expression is frequently compared with non-expressed/non-protein coding transcripts which is not a relevant comparison. CIT and CCNE1 “unaltered transcripts” are not protein-coding and it is unclear at what TPM they are expressed in these cells (the authors mention that in some cases they aren’t expressed at all). The authors mention that this is a control for the specificity of this approach, but their inclusion as a comparison thus does not make much sense to me and generally seems like a strawman comparison (eg. comparing any canonical TSS/transcript to a non- expressed/non-protein-coding transcript would technically show isoform-specific differences but not have any functional consequence).

- We agree with this definition in principle and have included the TPM values for all transcripts (Table S3) and have modified the text accordingly. We find that some of the transcripts (like CIT and CCNE1) are gained and that gain of expression results in a dependency. In the case of these two genes no other transcript is expressed (at least in the cell lines we tested). However, another class of transcripts (like MTA3 and ZFHX3) express more than one transcript however only one of these transcripts is essential for proliferation. In future studies we intend to ask whether these expressed transcripts may have different roles. Importantly, our previous studies have shown that proteins produced from these gained transcripts are presented on cell surface and could serve as potential immune markers (PMID: 28320776) which suggests that these are functional. Furthermore, here we show for the first time that for MTA3 and ZFHX3 a function for these transcripts. We have modified the text to better explain these differences. For each of these transcripts we compared both tumour vs normal and in addition within the tumour suppression of the altered vs un-altered transcripts. We agree with the reviewer that the definition of canonical is problematic and as such we have changed it throughout the manuscript to “unaltered transcript”. As now shown in the new Table S3 some of these are expressed while others are not. Furthermore, from the expression data alone it is hard to tell in some cases which is the P1 transcript (for

example HNF4A).

B) Three out of the four isoform-specific comparisons made in the paper seem to be just a GC-specific increase in the canonical TSS, which is obscured in the presentation of the data. With respect to canonical TSS identification, I agree with the authors that it can be difficult to ascertain based on various sources. However, another way of framing their isoform-specific CRISPRi screening approach is to answer the same question the authors pose in their manuscript: if someone used a common genome-wide CRISPRi library (ie. the CRISPRi Dolcetto library from the Doench Lab) would they have queried the ‘gained isoform’ the authors identified in their screen? For CIT/CCNE1/MTA1 the answer is yes, as all of the guides in this manuscript fall in the same region as the guides from the Dolcetto library. This indicates that the “isoform-specific differences” that the author mention for these genes are largely just the canonical transcript being turned on in the disease state (and there is no evidence that other transcripts are expressed at relevant levels). However, for ZFHX3 the answer is that Dolcetto only examines the P1 transcript guides and not the P2 transcript guides, highlighting a very interesting and important part of this approach; that the authors can examine non-canonical transcript usage in one screen.

- We added new expression data in Table S3. As is shown in the new table S3 almost 400 (half) of the transcripts we selected have at least one transcript with higher expression (which would be typically selected for in most common CRISPRi libraries). Furthermore, for many genes we find two transcripts with very similar levels which make it very difficult to determine which is the important one. This includes well known transcripts such as HNF4A. However, as the reviewer states this is still very important since it is still hard to predict 100% of expressed and important transcripts from expression data alone. As our data clearly shows in some cases more than one functional transcript is expressed from a gene and would be overlooked with most CRISPRi libraries.

As such the manuscript should be modified to reflect that the hits in CIT/CCNE1/MTA1 are likely from “canonical TSS” activation/transcription (and don’t really reflect alternative isoform usage), and that the results from ZFHX3 are from non-canonical alternative TSSs that become activated in gastric cancer. I would argue that only the ZFHX3 case truly reflects the title and abstract of this paper, while the

CIT/CCNE1/MTA1 cases are just cases involving the canonical isoform being turned on without any significant influences from the alternative isoforms. Importantly, this does not mute the impact of the CIT/CCNE1/MTA1 work in the paper as they are still interesting and relevant hits, however the isoform-specific usage story for these transcripts needs to be clarified and corrected in revision of the text/abstract.

- We have modified this accordingly. Also, we have modified the wording from canonical and non-canonical to altered and unaltered.

Issue #2: The authors provide no extra validation of the core tenet of their approach that I mentioned in my first review, which was that perturbations of alternative TSSs for different isoforms from the screen via CRISPRi does not also effect the expression of the main isoform of the given gene. While they performed this experiment in Figure #1 for HNF4A, these dCas9 perturbations are loci-specific and need to be repeated for other transcripts found in the screen. In response to this original query, the authors mentioned that they were unable to detect certain transcripts, but this point only confuses me further. The authors quantified ZFHX3 P1 expression in the first version of this manuscript (which is now removed in this revision) in addition to ZFHX3 P2 isoform expression. RNA-seq data from both TCGA and this manuscript (in the supplement) suggests that both ZFHX3 transcripts should be relatively evenly expressed and present in detectable amounts. So I am still unclear why the authors can’t use a healthy or diseased gastric line with ZFHX3 P1 expression for this validation. I state this point again because I am not sure whether the P2 transcript can be perturbed without effecting the P1 transcript (due to P2 being the shorter transcript), as many studies have shown that intronic dCas9 binding can reduce transcript levels in a strand-specific fashion by displacing RNAP2 (Qi+ Cell 2013, for example).

- As requested by the reviewer we have added this data for ZFHX3 and MTA3 (additional data 4C and 6) since the unaltered transcript from CIT and CCNE1 are not expressed (see new table S3) in these cells it is impossible to do this experiment for these two genes. The issue we have had with the P1 isoform of ZFHX3 is that the promoter is extremely GC rich and despite making 10 different sgRNAs the only ones

that suppressed P1 expression had multiple alignments to the genome. We did see the expected phenotype and gene suppression using those sgRNAs but due to the obvious issues off target effects (which were justifiably flagged by the reviewer) we did not include these results in the manuscript. However, for MTA3 we did this exact experiment and similar to HNF4A we could suppress either the P1 or the P2 transcripts.

As a side-note, the authors might want to highlight in the discussion that the extended N-terminus of the ZFHX3 protein P2 isoform may represent an opportunity for different mutational control during cancer that could potentially play a role in. the shift of TSG functionality to oncogenic functionality.

- We thank the reviewer for this comment and have added additional paragraph in the discussion.

Issue #3: The authors run into the same issue with sgRNA specificity with the newly-included MTA3 results, where sgMTA3_P2_2 has multiple off-targets including one with a 100% match on chr17. This worries me as the authors mentioned in their response to reviewers that they removed any guides that had multiple exact matches in this revision, and as such this guide shouldn’t even be included in the analysis.

- Multiple sgRNAs targeting MTA3 show this phenotype. Furthermore, we show that these sgRNAs are specific and effective in suppressing MTA3 expression. Even after removing this guide from the analysis we get a similar result.

Issue #4: I am unsure if it is completely appropriate to perform statistical comparisons on samples that have n=2 (and technically n=1 for each guide used in Figure#4H), though I will leave this point up to the editor’s discretion and statistical guidelines of the journal.

- We have added to all figure legends the exact number of experiments.

Small points to correct/add:

There are currently no text labels/legends for the supplemental tables

- We have added a legend to the new supp table file and added a separate legend.

Supplemental Table 5/6 are missing.

- We are not sure why…. We have combined all the supp tables into one file.

All bar graphs should ideally show data points, as some of the figures have shown

these while others have not.

- This has been modified as requested.

Figure #2H-M is not colorblind friendly

- We thank the reviewer for that comment. We have modified all the figures to be

colour blind friendly.

Figure #3C-E tpyo for “unaltered”

- We thank the reviewer and have corrected the typo.

Mentions of the “ZFHX3 promoter” in the text without references to which isoform.

- We are sorry and have modified this.
